# Interleukin 4 induces rapid mucin transport, increases mucus thickness and quality and decreases colitis and *Citrobacter rodentium* in contact with epithelial cells

**DOI:** 10.1080/21505594.2019.1573050

**Published:** 2019-01-21

**Authors:** S. Sharba, N. Navabi, M. Padra, J. A. Persson, M. P. Quintana-Hayashi, J. K. Gustafsson, L. Szeponik, V. Venkatakrishnan, Å. Sjöling, S. Nilsson, M. Quiding-Järbrink, M. E. V. Johansson, S. K. Linden

**Affiliations:** a Department of Medical Biochemistry and Cell Biology, Sahlgrenska Academy, University of Gothenburg, Gothenburg, Sweden; b Department of Microbiology and Immunology, Sahlgrenska Academy, University of Gothenburg, Gothenburg, Sweden; c Department of Microbiology, Tumor and Cell Biology, Karolinska Institutet, Stockholm, Sweden; d Department of Pathology & Genetics, Sahlgrenska Academy, University of Gothenburg, Sweden; e Department of Mathematical Sciences, Chalmer University of Technology, Gothenburg, Sweden

**Keywords:** Mucin, mucus, mucosa, *Citrobacter rodentium*, A/E pathogen, cytokine, IL-4, IFN-γ, colitis, host-pathogen interaction

## Abstract

*Citrobacter rodentium* infection is a murine model for pathogenic intestinal *Escherichia coli* infection. *C. rodentium* infection causes an initial decrease in mucus layer thickness, followed by an increase during clearance. We aimed to identify the cause of these changes and to utilize this naturally occurring mucus stimulus to decrease pathogen impact and inflammation. We identified that mucin production and speed of transport from Golgi to secretory vesicles at the apical surface increased concomitantly with increased mucus thickness. Of the cytokines differentially expressed during increased mucus thickness, IFN-γ and TNF-α decreased the mucin production and transport speed, whereas IL-4, IL-13, *C. rodentium* and *E. coli* enhanced these aspects. IFN-γ and TNF-α treatment in combination with *C. rodentium* and pathogenic *E. coli* infection negatively affected mucus parameters *in vitro*, which was relieved by IL-4 treatment. The effect of IL-4 was more pronounced than that of IL-13, and in wild type mice, only IL-4 was present. Increased expression of *Il-4, Il-4-receptor α, Stat6* and *Spdef* during clearance indicate that this pathway contributes to the increase in mucin production. *In vivo* IL-4 administration initiated 10 days after infection increased mucus thickness and quality and decreased colitis and pathogen contact with the epithelium. Thus, during clearance of infection, the concomitant increase in IL-4 protects and maintains goblet cell function against the increasing levels of TNF-α and IFN-γ. Furthermore, IL-4 affects intestinal mucus production, pathogen contact with the epithelium and colitis. IL-4 treatment may thus have therapeutic benefits for mucosal healing.

## Introduction

Enterotoxigenic *Escherichia coli* (ETEC) causes diarrhea through secretion of enterotoxins, whereas enteropathogenic *E. coli* (EPEC) and enterohaemorrhagic *E. coli* (EHEC) induce attaching and effacing (A/E) lesions on intestinal epithelial cells. *Citrobacter rodentium* is a mouse pathogen that uses the same mechanism as EPEC and EHEC to colonize epithelial cells. During the mid-point of infection, the host response to *C. rodentium* is primarily Th1/Th17 driven, whereas cytokines of “Th2/anti-inflammatory” type appear during clearance: interferon gamma (*Ifn-γ*) and interleukin (IL) *Il-12* become up-regulated throughout infection whereas *Il-4*, tumor necrosis factor alpha (*Tnf-α*) and *Il-6* mRNA become upregulated during clearance only [1].

Colonic mucus consists of two layers: an inner, firm, nominally sterile layer and an outer, loose layer, which is a niche for commensal bacteria [2]. Bacterial penetration of the inner mucus layer and access to the epithelium are important determinants of colitis, both in murine colitis models and in ulcerative colitis [3]. The highly glycosylated MUC2 mucin is the main component of colonic mucus and is secreted constitutively by goblet cells [4]. Components released from microbes (e.g. lipopolysaccharide) as well as factors produced by innate and adaptive immune responses can cause mucin discharge [4,5]. IL-13 induces goblet cell proliferation during *Trichinella spiralis* infection [6], and treatment with IL-13 secreting cells results in increased Alcian blue staining of acidic mucins in tissue of mice with asthmatic airway inflammation [7,8]. In contrast, simultaneous addition of IFN-γ and TNF-α to cultured cells render them devoid of mucus granules [9]. Thus, a Th1 type response (common to Gram negative bacteria such as *E. coli* and *C. rodentium*) is associated with parameters that could indicate a mucus decrease, whereas IL-13 has been linked to a mucin increase. However, neither mucin mRNA levels, nor the amount of mucin in the tissue, correspond unambiguously to mucus formation [10], since this depends on factors such as post translational regulation, mucin production, transport and secretion.

*C. rodentium* infection in mice lacking Muc2 results in high mortality, whereas wild type (WT) mice clear the infection spontaneously [11], and clearance is delayed in mice with defective mucus exocytosis [12]. *C. rodentium* bind to Muc2, and high numbers of bacteria are found among secreted Muc2 in infected animals, indicating that mucins may limit bacterial access to the epithelial surface or aid in transport of the pathogen from the epithelium [13]. The current knowledge indicates that the cytokine environment, IgG and mucins are important for eliminating A/E pathogens [14,15]. Cytokines affect mucin production in allergic reactions, worm infection and chronic infection [16–22], however, the mucus related events that occur during natural clearance of bacteria have yet to be elucidated. Here, we identified that the increased mucus thickness that occur during clearance of *in vivo C. rodentium* infection is accompanied by increased mucin glycoprotein production and the cytokine environment determined the mucus thickness during infection. The effects of the cytokines differentially expressed concurrently with increased mucus thickness on mucus related parameters were investigated *in vitro* in the presence and absence of *C. rodentium*, ETEC and EPEC infection. Together, these results indicated that the IL-4/signal transducer and activator of transcription 6 (Stat6) pathway was important for intestinal mucus production during infection, and finally we demonstrated a role of the IL-4/Stat6 pathway in mucosal healing, recovery of the mucus layer and removal of the pathogen from contact with the epithelium by stimulating versus inhibiting the pathway during *in vivo* infection.

## Methods

### Ethics statement

All experimental procedures were approved by the Göteborgs Djurförsöksetiska Nämnd (Ethic No. 261/09 and 57–2016) based on the regulation from Djurskyddsförordningen DFS 2004:4. The ETEC and EPEC strains have been deposited at the ETEC culture collection of University of Gothenburg and in the group of Å. Sjöling. Permission to use the strain collection was granted by the Regional Ethical Board of Gothenburg, Sweden (Ethics Committee Reference 088–10). All samples were anonymized.

### Animals

**Figure 1.**
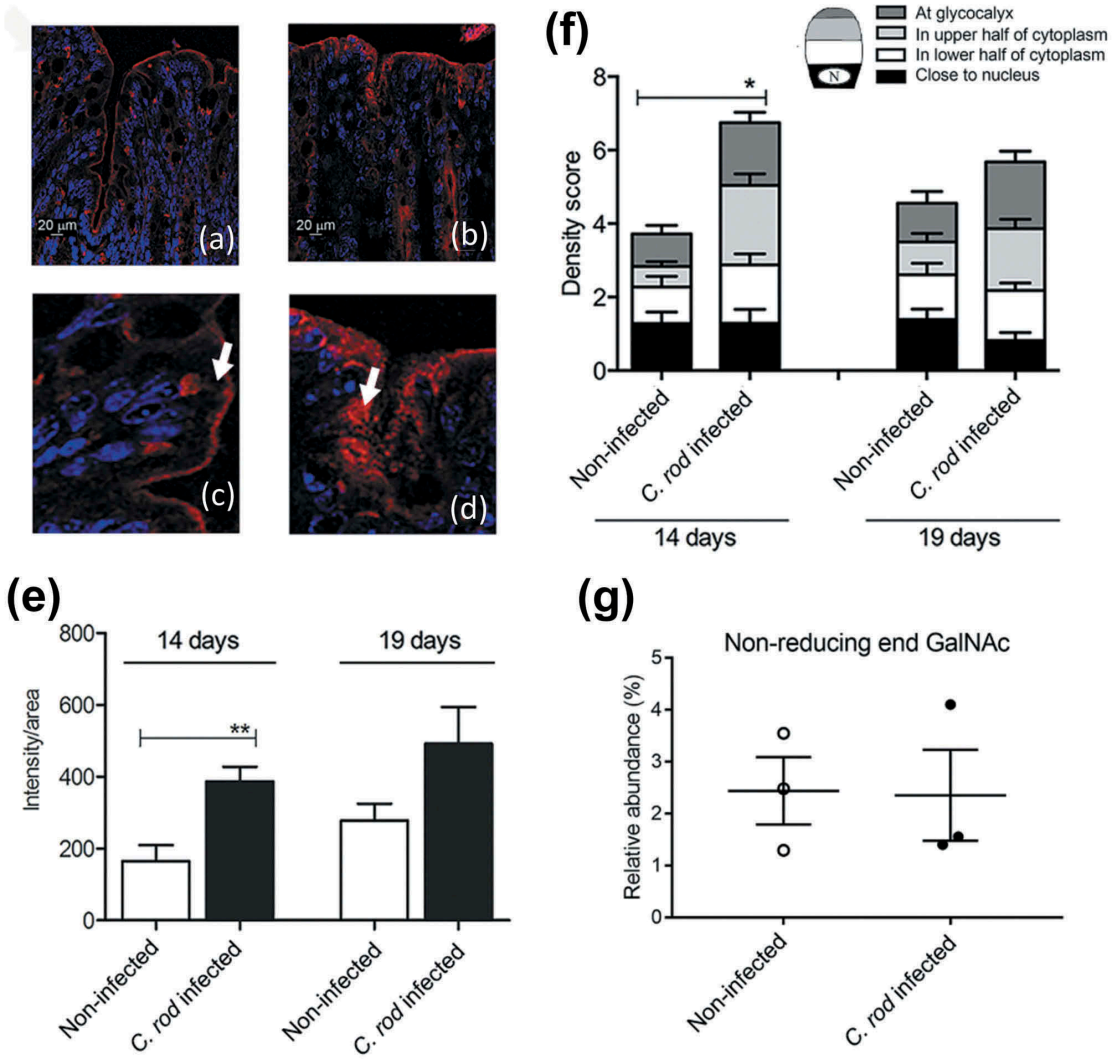
Mucin production/transport in the mouse colon during clearance of *C. rodentium* infection. (a-d) Incorporated GalNAz to mouse distal colon 3 h after intraperitoneal injection, TAMRA (red) and DAPI (blue). (a) non-infected and (b) *C. rodentium* infected mice harvested 14 days after infection using a 20x objective, (c) close-up of goblet cells from the same non-infected and (d) infected mice using 40x objective. Arrows point to the upper part of the goblet cell theca, which in the non-infected animals is devoid of GalNAz labelled mucin, whereas in the infected mice incorporated label is abundant. (e) Quantification of the incorporated TAMRA fluorescence intensity/area in the epithelial surface and neck of the distal colon of WT mice, **p < 0.01,: Mann-Whitney U-test, n = 6–8 day 14 and 3–4 day 19. (f) Blinded visual semi-quantification of the intensity of incorporated GalNAz in goblet cells, each location received a score of 0–4 based on intensity, *p < 0.05,: Mann-Whitney U-test, n = 6–8 day 14 and 3–4 day 19. (g) Quantification of the non-reducing end GalNAc among non-infected control mice and mice infected with *C. rodentium* for 14 days. Released *O*-glycans from distal intestine were analyzed on PGC-LC-MS/MS. Molecular mass, retention time on PGC column and tandem mass spectra along with in-house tandem mass spectral glycan library were used for structural identification (n = 3).

**Figure 2.**
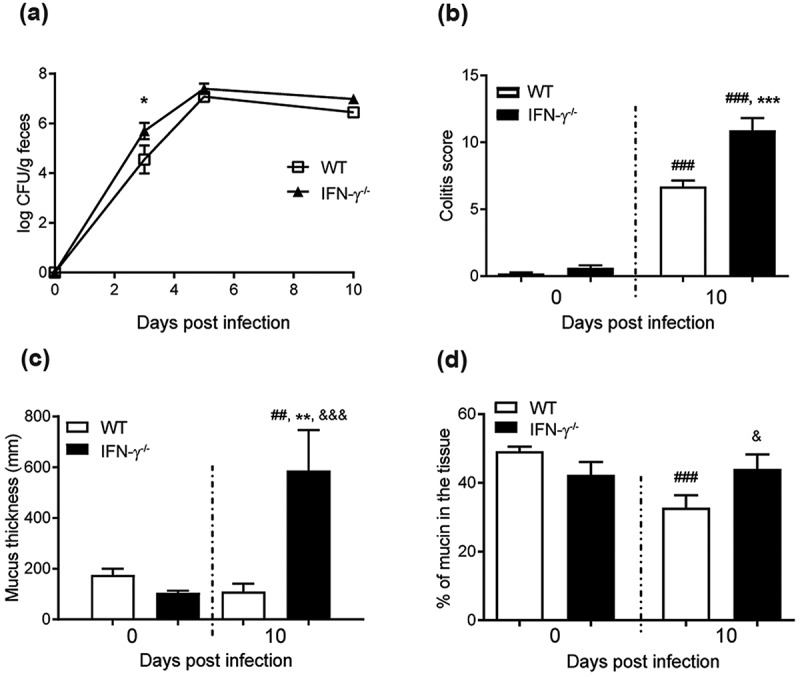
*C. rodentium* infection parameters, intracellular mucin storage and mucus thickness in WT and IFN-γ^−/-^ mice. (a) Colony forming units (CFU) of *C. rodentium* were analyzed in fecal pellets collected from individual mice. Compared to the non-infected mice, the number of *C. rodentium* increased in both WT and IFN-γ^−/-^ mice after infection. The CFU counts only differed between the groups at day three, and were indistinguishable at day five and 10 pi (n = day 0: IFN-γ^−/-^ 10 and WT 4, days three and five: 3–4, day 10: IFN-γ^−/-^ 16 and WT 4). (b) Colitis scores for WT and IFN-γ^−/-^ mice during infection. The score is calculated from the sum of crypt architecture, increased crypt length, emptied goblet cells, leukocyte infiltration, presence of lamina propria neutrophils, crypt abscesses, and epithelial damage and ulceration (Score 0–4/parameter) (n = day 0: IFN-γ^−/-^ 9 and WT 26, day 10: IFN-γ^−/-^ 10 and WT 19). (c) The thickness of the inner mucus layer in distal colonic explants of WT and IFN-γ^−/-^ mice in the non-infected animals at day 0 and 10 pi (n = IFN-γ^−/-^ 4 and WT 7) assessed by measuring the distance between the top of the mucus layer (visualized by addition of activated charcoal particles) and the epithelial surface using a micropipette connected to a micromanipulator. D) The percentage of the mucosa positive for AB/PAS staining in the colon of the WT and IFN-γ^−/-^ mice (n = IFN-γ^−/-^ 9–10, WT day 0: 21 and day 10: 18). Statistics: one way ANOVA, Holm-Šídák’s post hoc test, ##p < 0.001, ###p < 0.0001 vs day 0 of the WT mice, *p < 0.05, **p < 0.01, ***p < 0.0001 vs day 0 of the IFN-γ^−/-^ mice and &p < 0.05, &&&p < 0.0001 vs day 10 of the WT mice.

For the experiments shown in Figures 1, 2 and 6, 8–12-week old, specific-pathogen-free, male C57BL/6 (Charles Rivers, Germany) and IFN-γ-deficient (IFN-γ^−/-^) [23] mice on a C57BL/6 background, were bred in ventilated cages under pathogen-free conditions at the Laboratory for Experimental Biomedicine at Sahlgrenska Academy, Gothenburg University (Gothenburg, Sweden). For the remainder of the experiments, 8-week old male C57BL/6 mice were purchased (Charles Rivers, Germany) and housed under pathogen-free conditions at the Department of Rheumatology and Inflammation Research, University of Gothenburg (control/IL-4/Stat6 cohort 1) or in individually ventilated cages at the Laboratory for Experimental Biomedicine, Gothenburg University (control/IL-4/Stat6 cohort 2). The animals had a 12 h light/dark cycle, free access to water and food throughout the experiment and were monitored daily for the duration of the study.

### Infection and treatments in vivo

*C. rodentium* strain ICC169 grown on MacConkey agar (CM0007, Oxoid) for 20 h at 37°C were suspended in Luria-Bertani broth to an OD_600_ of 0.8. Male C57BL/6 mice were infected with 5 × 10^9^ CFU by oral gavage.

*Incorporation of GalNAz*: A total volume of 0.5 ml per mouse of GalNAz (C33365, Invitrogen) was prepared by dissolving 2.6 mg of GalNAz in 50 µl dimethyl sulfoxide (DMSO, A3672, AppliChem) and diluted in phosphate buffered saline (PBS) (0.15 mol/L NaCl, 5 mmol/L Na_2_HPO_4_ buffer, pH 7.4). The GalNAz injections were given intraperitoneally and mice were sacrificed 3 h after the injection.

*Stat6 inhibitor and IL-4 treatment*: The control/IL-4/Stat6 cohort 1 contained 4–5 mice in each treatment group, harvested at 14 days post infection (dpi) whereas the control/IL-4/Stat6 cohort 2 contained 5–6 mice in each treatment group for each harvest time point (13 and 22 dpi). Mice received either recombinant IL-4 (0.2 µg/mouse, I1020, Sigma-Aldrich, dose determined after a pilot experiment suggested that both 2 ng and 2 µg/100g mouse weight had an effect and the dose range for the pilot was determined previously [24,25]), Stat6 inhibitor (AS1517499, 1992, Axon, 10 mg/kg body weight, dose in accordance with a previous study [26]), or a second inhibitor acting downstream of Stat6 (RCM-1, GLXC- 10,156, Glixx Laboratories, 1.7 mg/kg body weight, dose in accordance with a previous study [27]) dissolved in sterile PBS containing 20% DMSO (A3672, AppliChem) and 1% bovine serum albumin (BSA, Sigma-Aldrich) for protein stabilization. Control mice received sterile PBS containing 20% DMSO and 1% BSA (vehicle). Treatments were administered by intraperitoneal injections in a volume of 0.1 ml for three consecutive days (for cohort 1: from day 11 to day 13, cohort 2: day 10 to day 12 pi) and mice harvested at day 22 received two additional injections on day 15 and 17 pi. In the results section, we show data from the AS1517499 Stat6 inhibitor. To verify the results with the AS1517499 Stat6 inhibitor, we treated four additional mice harvested at day 13 pi with a second inhibitor (RCM-1), and the parameters bacterial localization, density and colitis scores matched those from the AS1517499 treatment.

*Harvest*: mice were anesthetized with isoflurane and sacrificed by CO_2_ and cervical dislocation. Starting at the anal verge, colon samples were collected. The most distal 1–2 cm colonic specimens were collected in ice cold transport KREBs buffer for mucus thickness measurements. The second distal 0.5 cm of colon was harvested into 0.5 ml fresh Carnoy’s methanol fixative (60% dry methanol, 30% chloroform, 10% glacial acetic acid) and the next 0.5 cm into 200 µl RNAlater (AM7020 Thermo Fisher Scientific). For cohort 2, the next 1.5 cm was harvested for flow cytometry.

### qPCR

Distal colon samples (0.5 cm) were collected in RNAlater and kept at 4ºC overnight, then at −80ºC. For the analysis of mucin genes, total RNA was extracted using the RNeasy mini kit (74,104, QIAGEN following the manufacturer’s protocol. The QuantiTect Reverse Transcription kit (20,531, QIAGEN) was used for cDNA preparation from extracted RNA of each sample, according to the manufacturer’s protocol. SYBRGreen based real time qPCR was carried out using primers for *Muc1, Muc2, Muc4, Muc6, Muc13, Clca3*, using previously published primers [10]. Data were normalized with the *Hprt-1* housekeeping gene from QIAGEN. Fold changes were calculated using the ΔΔCT method with the mean of CT from three non-infected mice as control. Fold changes ≥4 were accepted as up-regulation and ≤0.25 as down-regulation. Murine gastric tissue was used as a positive control for *Muc1*. For the qPCR cytokine array, mRNA from two sets of two mice in each group were pooled for the time points of day 0 and day 10 (i.e. data are representative of four mice in each group), whereas the time points day 14 and 19 contained mRNA pooled from three mice in each group. The QuantiTect Reverse Transcription kitwas used for cDNA preparation according to the manufacturer’s protocol. qPCR on pooled samples was carried out in duplicates using RT^2^ Profiler™ PCR Array (PAMM-034Z, QIAGEN) plates containing 84 mouse inflammatory cytokines, chemokines and receptor genes. The array plates were run on an ABI 7500 real-time PCR system (Applied Biosystems). Intra-plate controls were included and data were normalized by the RT^2^ Profiler™ PCR Array data analysis software (QIAGEN) using the most suitable housekeeping gene chosen from five housekeeping genes (*Gusb, Hprt1, Hsp90ab1, Gapdh, Actb*) with a threshold of variance of only 0.2 cycles. The means of two technical replicates were used to calculate fold changes against control mice using the same software. Fold changes ≥2.5 were accepted as up-regulation and ≤0.4 as down regulation, in compliance with instructions from the array manufacturer. Protein interactions were analyzed by the database and web-tool STRING v10 (Search Tool for the Retrieval of Interacting Genes/Proteins) [28]. The interactions include both physical and functional associations derived from numerous sources, including experimental repositories and public text collections. To verify the cytokine array results and pathways involved in mucin regulation during infection with individual qPCRs, total RNA was extracted from an additional three non-infected WT control mice and three mice from 19 dpi using TRizol (15596018, Thermo Fisher Scientific). RNA purity was assessed through UV spectroscopy (NanoDrop; Thermo Fisher Scientific). Total RNA (for cytokines 5 μg, and pathways 7.5 μg) was treated with DNase at 37°C for 30 min, addition of 5mM ethylenediaminetetraacetic acid (EDTA) and heat inactivation of DNase at 75°C for 10 min followed by cDNA synthesis. A final concentration of 5 mM MgCl_2_ was added to RNA samples, which were later used for cDNA synthesis by adding oligo(dT) primers and Superscript III (18080093, Thermo Fisher Scientific) at 50°C for 2 h. The cDNA was used in a qPCR using EvaGreen (172520, Bio-Rad Laboratories) with *Il-4, Tnf-α, Ifn-γ Stat6, Spdef, Myd88* (QIAGEN) and *Nfκb1* (Fwd: GAACGATAACCTTTGCAGGC, Rev: TTTCGATTCCGCTATGTGTG); designed using Primer depot https://mouseprimerdepot.nci.nih.gov/cgi-bin/testdb2.pl) primers. The expression of *Hprt1* (QT00166768, QIAGEN) was used for normalizing the qPCR data. Samples were amplified in triplicate with a negative control without reverse transcriptase to confirm the lack of contaminating genomic DNA. Data acquisition and analysis were performed using CFX manager 3.1 software (Bio-Rad Laboratories).

### Mucus measurements

Mucus thickness comparisons between WT and IFN-γ^−^^/-^ mice:

Following incubation on ice for 30 min, the longitudinal muscle was removed and the tissue was mounted in the perfusion chamber. The apical and basolateral solution was KREB’s buffer (115.8 mM NaCl, 1.3 mM CaCl_2_, 3.6 mM KCl, 1.4 mM KH_2_PO_4_, 23.1 mM NaHCO_3_, 1.2 mM MgSO_4_, Merck) containing 5.7 mM Na-Pyruvate, 5.1 mM Na-L-Glutamate and 10 mM D-Glucose for the basolateral compartment, whereas the apical compartment was immersed in KREB’s buffer containing 5.7 mM Na-Pyruvate, 5.13 mM Na-L-Glutamate and 10 mM D-Mannitol. The solutions were gassed with 95% O_2_ and 5% CO_2_ prior to the experiment and pH was set to 7.4. The perfusion chamber was heated to 37°C and kept at a constant temperature throughout the whole experiment. Tissue viability was measured using transepithelial potential difference (PD) recordings. To visualize the colonic mucus layer a suspension of activated charcoal particles was added to the apical surface and allowed to sediment down to the top of the mucus layer. The thickness of the mucus layer was assessed by measuring the distance between the top of the mucus layer, and the epithelial surface using a micropipette (tip diameter, 5–10 µm) connected to a micromanipulator.

### Ex vivo imaging of mucus in WT infected mice with and without IL-4 treatment

Live imaging of colonic explants was performed as described previously [29,30], with slight modifications. Briefly, 1.5 cm of distal colon was removed by dissection and flushed with ice cold KREB’s buffer. The tissue was cut open along the mesentery border, with the muscle layer kept intact, and mounted in a horizontal perfusion chamber. Staining of the tissue was performed by 10 min apical incubation of 200 µl Calcein violet (C34858, Invitrogen; 6 µg/ml) in KREB’s-Mannitol. Thereafter, 10 µl of FluoSphere Carboxylate-Modified Microspheres 1 µm (F8816, Invitrogen; 1:10) in KREB’s-Mannitol was added and left to sediment for 5 min. Excess beads were removed by gentle washing prior to confocal imaging. Four Z-stacks per animal was acquired with an LSM700 Examiner Z.1 upright confocal microscope with Plan-Apochromat x20/1.0DIC water objective and Zen imaging software (Zeiss). Mucus penetrability was assessed based on the number of beads in contact with the epithelium. Beads were counted manually in the combined tissue area of all four Z-stacks (0.4 mm^2^ per animal) using Volocity 5.5.1 software (Perkin-Elmer). Mucus thickness was calculated as the average distance between beads and epithelium. The Z-positions were extracted by mapping tissue and bead Isosurfaces in Imaris Software (Bitplane). A flat tissue area of 150 × 150 µm in XY-plane from three Z-stacks per animal was used for the analysis.

### In vitro mucosal surfaces

HT29 MTX-E12 cells (derived from MTX selected colorectal adenocarcinoma cells HT29, ATCC) were cultured in RPMI medium (BE12-702F/U1, Lonza) containing 10% (V/V) fetal bovine serum (FBS), 1% (V/V) penicillin G sodium and streptomycin (DE17-603E, Lonza). The cells were plated on snapwell tissue culture inserts (0.4 μm pores, 1.12 cm in diameter, 3407, Corning) at a density of 7.5 × 10^4^ cells/well under standard conditions (media added: 4 ml to the basolateral and 200 µl to the apical compartment) until complete confluency. The cells were then changed to semi-wet interface with mechanical and chemical stimulation as described previously [31,32]. Briefly, the basolateral and apical media were changed to 2 ml and 50 µl respectively, and 10 µM of the Notch γ-secretase inhibitor (DAPT, D5942, Sigma-Aldrich) was added to the basolateral media during the first 6 days of semi-wet interface, and plates were placed on a rocking board at 37ºC, 5% CO_2_, atmospheric O_2_ for 34 days.

*In vitro cytokine treatment*: For cytokine treatment and infection studies cell culture media were changed into antibiotic-free RPMI (BE12-702F/U1, Lonza) with 10% FBS at day 30 post confluency, and designated concentrations of different cytokines were added to the basolateral media. The concentration of cytokines used in the cell treatment is presented in Table 1. The concentration of each cytokines was chosen based on previous work that was either related to immune responses, mucin production or the non-destructive effect on the *in vitro* mucosal surface [33–38].

**Table 1.** Concentration of cytokines used to treat the *in vitro* model. The concentrations of the human cytokines were chosen based on previous work either related to immune response/mucin production and/or lack of detrimental effect on cell lines [33–38].

Cytokine	Concentration
Interferon gamma (IFN-γ)	10 ng/ml
Tumor necrosis factor alpha (TNF-α)	10 ng/ml
Interleukin 4 (IL-4)	1.5 ng/ml
Interleukin 6 (IL-6)	15 ng/ml
Interleukin 12 (IL-12)	20 ng/ml
Interleukin 13 (IL-13)	1.5 ng/ml
Interferon gamma (IFN-γ) + Tumor necrosis factor alpha (TNF-α)	10 ng/ml+10 ng/ml
Interferon gamma (IFN-γ) + Tumor necrosis factors alpha (TNF-α) + Interleukin 4 (IL-4)	10 ng/ml+10 ng/ml+1.5 ng/ml

*In vitro incorporation of GalNAz*: 5.2 mg of GalNAz was dissolved in 1 ml DMSO and diluted in 250 ml of culture media (RPMI with 10% FBS) without antibiotics. Then 1 ml of the basolateral media of each well was replaced with media containing GalNAz. The membrane was collected in Methanol Carnoy’s fixative 2 h after addition of GalNAz.

*In vitro infection: C. rodentium* strain ICC169, ETEC strain number E2265 (isolated from an adult patient with diarrhea in Dhaka, Bangladesh [39] and EPEC strain 38068 (positive for eae and bfp, Culture Collection, University of Gothenburg, Sweden), were grown on MacConkey agar for 20 h at 37°C, then harvested from the plate in sterile PBS. For infection, 10 µl of *C. rodentium* and *E. coli* suspensions with a respective OD of 2.0 and 0.1 at 410 nm (CFU: 1 x 10^7^ and 5 × 10^5^ CFU, corresponding to a multiplicity of infection of 10:1 and 0.5:1) in sterile PBS was added to the apical side of the HT29-MTX-E12 based *in vitro* mucosal surfaces 24 h prior to collecting them in Carnoy’s methanol fixative.

### Histological stains

PAS/Alcian blue stain: deparaffinized sections were immersed in 100% ethanol for 10 min, rinsed in water for 10 min, immersed in 3% acetic acid for 2 min and stained in 1% Alcian Blue (AB) 8GX (A5268, Sigma-Aldrich) in 3% acetic acid (pH 2.5) for 2.5 h. Non-specific stain was removed with 3% acetic acid and sections were rinsed in water for 10 min. The slides were then oxidized in 1% periodic acid in water at room temperature for 10 min, washed in water for 5 min, immersed in Schiff’s reagent (3,952,016, Sigma-Aldrich) for 10 min, rinsed in water for 5 min and then incubated three times in 0.5% sodium meta-bisulfite before a final wash in water. ImageJ was used to quantify the percentage of tissue that stained positive for AB/PAS in the total distal colon area. ImageJ is a public domain image processing program (Wayne Rasband at the Research Services Branch (RSB) of the National Institute of Mental Health (NIMH), National Institutes of Health, Maryland, USA). Morphology of the cell lines was scored in a blinded fashion at 20x magnification and is expressed as the sum of the individual scores for number of crypt like structures (0–3), integrity of the epithelial layer (0–3) and AB/PAS positive material in the surface cells (0–3). The total section of undamaged membrane was scored; for treatments that severely affected the membranes (i.e. ETEC infection in combination with TNF-α and IFN-γ), large parts of the membrane and sometimes the entire membrane was lost during processing, leading to the presented scores most likely underestimating the level of damage, as the most damaged parts were the parts that became more fragile and lost. Furthermore, this resulted in a low n for those treatments.

For the total colitis score of infected mice, tissue sections were stained with haematoxylin/eosin (H/E) and slides were coded to blind the analysis. The full section was systematically scored for: crypt architecture (0–4), tissue damage (0–4), crypt length (0–4), neutrophil count in lamina propria (0–4), inflammatory cell infiltration (0–4) and crypt abscesses (0–4). For the IL-4/Stat6 cohorts, the crypt length scores were excluded due to the fact that we did not have fecal pellet-free sections available for histology for all mice, which is needed for accurate measurements. However, for the sections available, the crypt length scores followed the same trend as the other colitis sub-scores for each treatment group.

### Immunofluorescence

Paraffin embedded sections were deparaffinized and rehydrated. For antigen retrieval, sections were heated in 0.01 M citric acid buffer pH 6 for 10 min or 30 min at 99°C for cells and tissues respectively. Non-specific binding was blocked by 5% FBS and the sections were incubated with an antibody recognizing the murine and human Muc2/MUC2 mucin (polyclonal rabbit-anti-MUC2C3 [2], kind gift of Professor G. Hansson, University of Gothenburg, Sweden) and MUC5AC (monoclonal mouse-anti-MUC5AC, 45M1, Sigma-Aldrich) diluted (1:1000) in blocking buffer for 2 h at RT. Slides were then incubated for 1 h at RT with Alexa Fluor 488-conjugated anti-rabbit and Alexa Fluor 594 anti-mouse immunoglobulins (1:500, Thermo Fisher Scientific), respectively. For bacterial localization, sections were co-incubated with monoclonal rabbit-anti-*E. coli* O152 antiserum (295,774, Denka Seiken) diluted 1:100 and FITC-conjugated Maackia amurensis lectin II (MAA II, to visualize mucins, F-7801–2, EY Laboratories) diluted to 25 µg/ml in Tris buffered saline (TBS, 50 mM Tris-Cl, 150 mM NaCl, pH 7.6) for 1h at RT. Tissue sections were rinsed three times in TBS and incubated for 1h at RT with Alexa Fluor 594-conjugated goat anti-rabbit (1:100) followed by three 5 min washes in TBS. To visualize DNA, the sections were mounted with DAPI containing Prolong Gold anti-fade reagent (P36935, Thermo Fisher Scientific). Pictures were captured with an Eclipse 90i fluorescence microscope (Nikon). Quantification of mucin in the *in vitro* model was done by measuring the integrated fluorescence density (the total intensity of fluorescence in the defined area) obtained by using the ImageJ program (Wayne Rasband). The percentage of goblet cells was counted in a blinded fashion at 40x magnification. For bacterial localization, the tissue section was scored based on O152 density in the inner mucus layer/in close association with epithelial cells (Score 1 = >10%, 2 = 10–50% and 3 = >50%) and the total number of crypts colonized from the neck or epithelial lining and down (Score 1 = 1–3 crypts, 2 = 3–5 crypts and 3 = >5 crypts). Bacterial density was scored in a blinded fashion at 40x magnification.

### Fluorescence in situ hybridization

The slides were deparaffinized and dehydrated using increasing concentrations of ethanol. Hybridization solution (40% (v/v) formamide, 0.1% sodium deodecyl sulfate, 0.9 M NaCl, 20 mM Tris-HCl pH 7.4) containing 10 ng/µl of Cy3.5 5´ labeled eubacteria-specific probe (5´GCTGCCTCCCGTAGGAGT-3´) was added to tissue sections and incubated at 37°C in a humidified chamber containing 40% formamide over-night. Subsequently, the slides were washed with 0.9 M NaCl, 20 mM Tris-HCl pH 7.4 at 50°C for 20 minutes, followed by a brief submersion in room temperature PBS. For co-staining, sections were incubated with monoclonal rabbit-anti-*E. coli* O152 antiserum (295774, Denka Seiken) diluted 1:100 at 4°C over-night followed by over-night incubation with Alexa Fluor 594-conjugated goat anti-rabbit diluted 1:100 rinses and reagents were used as mentioned above for immunofluorescence. Cell mask (C10046, Thermo Fisher Scientific) diluted (1:14000) in PBS was applied and sections were incubated for 30 minutes at room temperature followed by a brief wash in PBS and one in distilled H_2_O. DAPI (P36935, Thermo Fisher Scientific) was used to visualize DNA as described above. Pictures were captured with an Eclipse 90i fluorescence microscope (Nikon).

### Fluorescent detection of GalNAz

Paraffin embedded samples were deparaffinized, hydrated, and washed in PBS. Membrane sections were incubated with 20 µl of the reaction mix from the tetramehylrhodamine (TAMRA) glycoprotein detection kit (C10410, Thermo Fisher Scientific) and incubated at room temperature for 2 h, followed by wash in PBS. The sections were mounted with DAPI containing Prolong Gold anti-fade (P36935, Thermo Fisher Scientific) reagent to visualize DNA (nuclei). The intensity and localization of freshly produced mucins were evaluated in a blinded fashion at 40x magnification on an Eclipse 90i fluorescence microscope (Nikon). Due to that co-stain with antibody against Muc2 affected the TAMRA intensity, goblet cells were identified based on cellular morphology.

### Evaluation of igg levels in serum by enzyme linked immunosorbent assay (ELISA)

Serum was obtained from mouse blood samples by centrifugation at 3000 x *g* for 10 min at 4°C. *C. rodentium* was grown as mentioned above, harvested in sterile PBS, washed once and sonicated 5 × 15 seconds with a 3 min interval between sonications. The suspension was then centrifuged at 4000 x *g* for 4 min at 4°C, the supernatant collected, diluted to an OD_280_ of 0.4 and incubated in Polysorb ELISA plates (735–0131, Thermo Fisher Scientific) at 4°C over-night. The plates were washed 3 x in PBS with 0.05% Tween-20 (PBS-t) and unbound sites were blocked for 1 h in blocking solution (PBS containing 1% BSA). Samples were then plated in triplicates incubated with 100 µl/well murine sera (diluted 1:100, 1:1000, 1:10000 and 1:100000 in blocking solution containing 0.05% Tween-20) for 1h at RT. Plates were then washed three times in PBS-t and incubated with 100 µl/well HRP conjugated goat-anti-mouse IgG (115–035-209, Jackson ImmunoResearch) diluted 1:2000 in blocking solution. Plates were washed three times in PBS-t and bound secondary antibody was visualized using Tetramethylbenzidine substrate reagent (T0440, Sigma-Aldrich). The reaction was allowed to proceed for 5 min and then stopped by adding 100 µl of 0.5 M H_2_SO_4_. Absorption at 450 nm was measured (Wallace Victor2, 1420 Multi-label counter, Perkin-Elmer) and background control (signal in wells without bacterial lysate) was subtracted from the test absorbance.

### Flow cytometry analyses

Cells from Mesenteric lymph nodes (MLNs) were extracted by mashing through a 50 µm filter. Colon samples of 1.5 cm were harvested as mentioned above for cell extraction from the lamina propria. For colonic analyzes, two mice per sample were pooled resulting in one non-infected control sample and two infected samples. Colonic tissue was processed as described earlier [40], but with the use of collagenase VIII (C2139, Sigma-Aldrich) for digestion. Briefly, colon tissue was cut into ~5 mm pieces and washed in EDTA (15575020, Thermo Fisher Scientific) buffer to remove epithelial cells. Tissue was then digested with collagenase and the remaining pieces were mixed with gentle MACS dissociator. Collected cells from colons and mesenteric lymph nodes were stimulated with 50 ng/ml of phorbol myristate acetate (P8139, Sigma-Aldrich) and 500 ng/ml of ionomycin (I3909, Sigma-Aldrich) at 37°C for 5 hrs in the presence of Golgiplug (555029, BD Biosciences). Cells were stained with LIVE/DEAD Fixable Aqua (L34957, Thermo Fisher Scientific), followed by surface staining with CD45-APC/Fire750 (clone 30.F11), TCRbeta-BUV737 (H57-597), CD4-AF700 (GK1.5) and CD8α-BUV395 (53–6.7) purchased from BD Biosciences and Biolegend. Intracellular staining of IL-4-Phycoerythrin (11B11, BD Biosciences) was performed with the FOXP3 staining kit (00–5523-00, ebioscience). Stained cells were run on Fortessa X20 (BD Biosciences) and analyzed in FlowJo.

### Mass spectrometry for GalNAc determination

*Cell lysis and protein extraction*: Murine colon tissues were homogenized and the cells lysed in 100 mM Tris-HCl pH 7.5, 150 mM NaCl, 1 mM EDTA, 1% Triton X-100, 10 mM dithiothreitol and protease inhibitor cocktail (Sigma Aldrich). Samples were incubated in ice for 15 min, followed by homogenizing twice for one min with 5 min pause between treatments. Protein extraction was carried out overnight at 4°C by end-over-end rotation, followed by centrifugation for 30 min, 12,000 RPM at 4°C. Supernatants containing the soluble extracts were collected and the protein concentrations were measured using NanoDrop 2000 (Thermo Fisher Scientific).

*Release of O-glycans from extracted proteins*: Approximately 100 µg of extracted protein samples were dot blotted on a PVDF membrane (Millipore). Proteins were stained using Alcian blue stain solution, excised and transferred to an Eppendorf tube. *N*-glycans were released from the intact proteins using 10 U *N*-glycosidase F (PNGase F, *Elizabethkingia miricola*, Promega) in 20 µl water/tube by overnight incubation at 37°C. The released *N*-glycans were not used further in this study. Subsequently, the samples were subjected to reductive β-elimination with 0.5 M sodium borohydride in 50 mM sodium hydroxide for 16 h at 50°C to release the *O*-glycans. The reduction reaction was quenched by the addition of glacial acetic acid to the mixtures and desalted using strong cation exchange resin packed on top of C18 column. The solid-phase extraction removed cations and any protein or peptide component remaining. Excess borate was extracted as methyl esters by repeated evaporation.

*Characterization of O-glycans*: Released *O*-glycans were analyzed by liquid-chromatography-tandem mass spectrometry (LC-MS/MS) using a 10 cm X 250 µm i.d. column (in-house), containing 5 µm porous graphitized carbon (PGC) particles (Thermo Fisher Scientific) connected to an LTQ mass spectrometer (Thermo Fisher Scientific). *O*-glycans were eluted using a linear gradient from 0 to 40% acetronitrile in 10 mM ammonium bicarbonate over 40 min at a flow rate of 250 nl/min. Electrospray ionization-mass spectrometry (ESI-MS) was performed in negative ion polarity with an electrospray voltage of 3.5 kV, capillary voltage of −33.0 V, and capillary temperature of 300°C. The following scan events were used: MS full scan (*m/z* 380–2000) and data-dependent tandem MS (MS/MS) scans after collision-induced dissociation (CID) on precursor ions at a normalized collisional energy of 35% with a minimum signal of 300 counts, isolated width of 2.0 *m/z*, and activation time of 30 ms. The data were viewed and manually analyzed using Xcalibur software (version 2.2, Thermo Fisher Scientific).

### Statistics

All tests were performed using Prism (GraphPad Software, version 3 · 0) or SPSS statistics 18 (IBM). Values are expressed as mean ± S.E.M or geometric mean ± interquartile ranges. Comparison of data between control and infected at a specific time-point was made using the unpaired *t*-test or Mann-Whitney U-test. One-way analysis of variance (ANOVA) with the Holm-Šídák’s or Dunnet’s multiple comparison or Kruskal-Wallis tests were used to compare data from more than 2 experimental groups. The analysis of fecal CFU counts was performed with a linear mixed effects model comparing the slopes of the three treatment groups.

## Results

### *The increased mucus thickness during* C. rodentium *clearance is accompanied by increased mucin production and speed of transport en route exocytosis* in vivo

During *C. rodentium* infection, the mucus layer initially becomes thin, as measured at 4 and 10 dpi, followed by an increase in thickness during clearance, as measured at 14 and 19 dpi [41]. The azide-modified galactosamine (GalNAz) incorporates into the core region of mucin *O*-glycans and can be used to analyze mucin production and transport [22,42,43]. Newly synthesized mucins can be visualized in the supranuclear compartment of the cell one hour after injection [22] and reach the colonic lumen in 6–8 h [44]. We analyzed quantity and cellular location of labeled mucins during clearance of infection at 3 h post GalNAz injection; because by that time the mucins are inside the cells and potential processing artefacts arising in the less stable mucus layer do not affect the analysis. The level of GalNAz incorporation in the epithelial surface cells doubled during clearance (p < 0.01, Figure 1(a-e)). The non-goblet cells produce membrane bound mucins such as Muc4, Muc13 and Muc17, and during *C. rodentium* infection Muc1 is induced [13], which likely contributes to the increased GalNAz incorporation. We quantified the location of the GalNAz labeled mucins in the goblet cells separately to investigate if increased Muc2 production could be a contributing factor to the increased mucus thickness. In non-infected mice, incorporated GalNAz mainly localized to the supranuclear area in the goblet cells, although some label was also detected apically (Figure 1(a, c and f)). The latter represents membrane bound mucins, which incorporate into the apical cell membrane faster than the large multimeric mucins release into the mucus layer [22]. In contrast to the non-labeled theca of goblet cells in non-infected mice, GalNAz was mainly found in the mucus theca 14 and 19 dpi (Figure 1(b, d and f)). Thus, incorporation and transport of newly synthesized GalNAz labeled mucins through the goblet cells to the secretory vesicles at the apical surface was faster in mice at 14 dpi compared to non-infected mice (p < 0.05, Figure 1(f)), and a similar trend remained at 19 dpi (p < 0.2). Although the vast majority of GalNAc are present in the linkage between the protein and the glycan, they can also be present in the terminal region. To ensure the increased amount of incorporated label was not due to changed glycosylation, we analyzed released *O*-glycans by liquid-chromatography-tandem mass spectrometry (LC-MS/MS). In total, 4 structures containing non-reducing end GalNAc were detected; 3 of those contained terminal blood group A antigen (GalNAcα1,3(Fucα1,2)Galβ) and the other one a LacDiNAc epitope (GalNAcα1,4GlcNAcβ). The relative abundance of these glycans was less than 4% in total and did not differ between infected and non-infected mice (Figure 1(g)).

### The cytokine environment has a direct impact on mucus thickness during in vivo infection

To investigate effects of the pathogen versus the cytokine environment on mucus thickness, we identified that IFN-γ^−/-^ mice had a similar pathogen burden at 10 dpi (Figure 2(a)), but increased *Il-13, Il-5, Il-4*, and *Il-6* in IFN-γ^−/-^ mice compared to WT mice [1]. IFN-γ^−/-^ mice had higher colitis scores (p < 0.001, Figure 2(b) and Supplementary Figure 1). In non-infected animals, the thickness of the inner adherent mucus layer was similar in WT and IFN-γ^−/-^ mice. Ten dpi, the thickness decreased by 20% in WT mice, whereas it increased by 400% in the IFN-γ^−/-^ mice (p < 0.01, Figure 2(c)). The infected WT mice had a decreased amount of stored mucin in the mucosa, whereas the stored mucin content in the IFN-γ^−/-^ mice was similar to pre-infection levels (Figure 2(d)). Thus, the cytokine environment had a stronger influence on the mucus thickness than the pathogen alone. Similarly to the mucus changes during different time points of infection in WT animals [10], *Muc1, Muc2, Muc4* or *Muc6* mRNA levels did not explain the differences in mucus thickness between IFN-γ^−/-^ and WT mice (Supplementary Figure 2). Mucins consist of 90% carbohydrates and mucin production requires a multitude of glycosyltransferases and is subject to posttranscriptional regulation. It is thus not surprising that there is no clear association between levels of mRNA coding for the protein part of the mucin and mucus thickness or mucin glycoprotein production. Analogous to the absence of correlation between mucin mRNA and mucus thickness found here, a 50% decrease in mucin mRNA levels has been reported concomitantly with a 3-fold mucin protein increase in the murine small intestine [45,46].

### IFN-γ and TNF-α decreased the integrity and mucin content of the in vitro mucosal surfaces, whereas IL-4 and IL-13 enhanced these aspects

**Figure 3.**
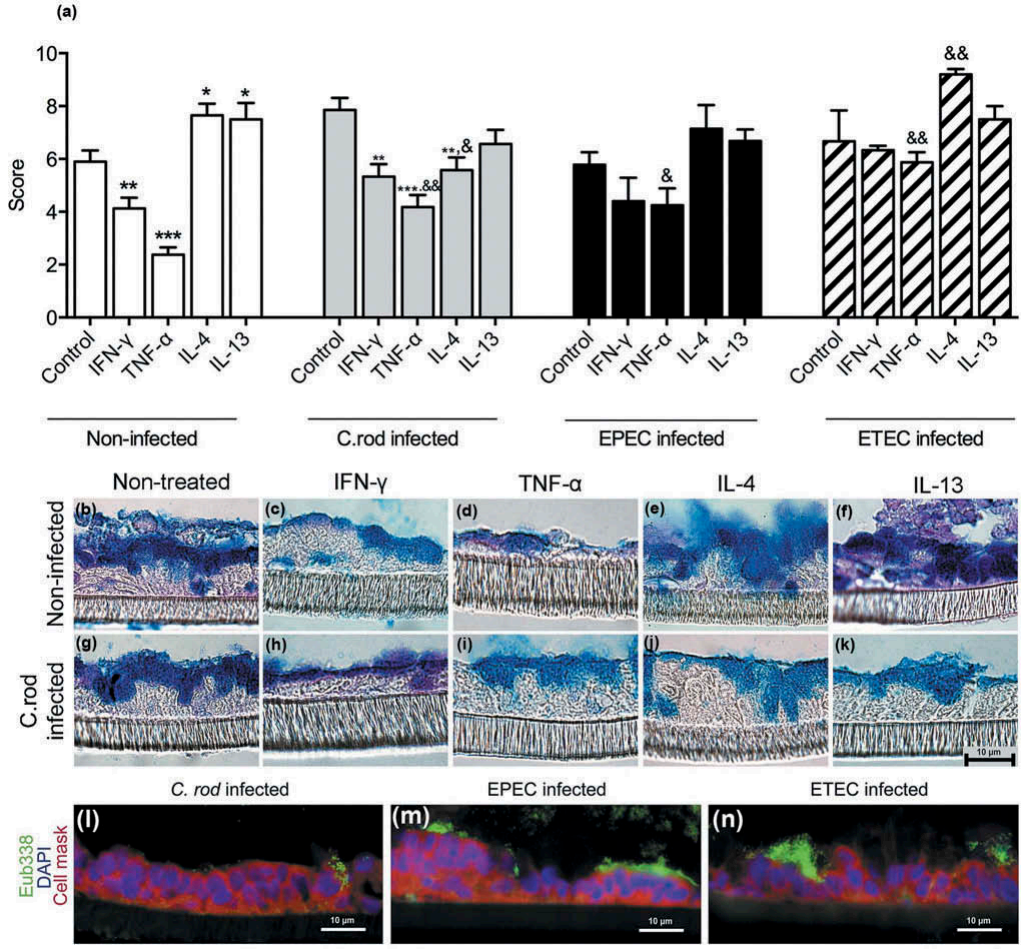
Morphology score of *in vitro* mucosal surfaces with and without *C. rodentium*, EPEC or ETEC infection. (a) Score reflecting the morphology (based on number of crypt like structures 0–4, integrity of the epithelial layer 0–4, and presence and mucus engorgement of surface goblet cells 0–4). Statistics: one way ANOVA, Dunnett’s post hoc test, *p < 0.05, **p < 0.001 and ***p < 0.0001 compared to controls in each group, and one way ANOVA, Holm-Šídák’s post hoc test, &p < 0.05, &&p < 0.001 compared to the non-infected *in vitro* mucosal surfaces treated with the same cytokine (n for non-infected: control 23, IFN-γ 12, TNF-α 12, IL-4 13, IL-13 11, for *C. rodentium* infected: control 9, IFN-γ 12, TNF- α 11, IL-4 13, IL-13 8, for EPEC infected: control 9, IFN-γ 5, TNF- α 6, IL-4 6, IL-13 5, for ETEC infected: control 5, IFN-γ 5, TNF- α 5, IL-4 5, IL-13 5), results are the mean of three experiments. B-K) AB/PAS staining of *in vitro* mucosal surfaces: B) Non-infected and non-treated, (c) treated with IFN-γ, (d) TNF-α, E) IL-4, (f) IL-13, (g) *C. rodentium* infected non-treated, (h) treated with IFN-γ, (i) TNF-α, (j) IL-4 and (k) IL-13. The *in vitro* mucosal surfaces were stained with Eub338 (green) and cell mask (red) to visualize bacterial localization in relation to the epithelial cells after 24 h of infection with (l) *C. rodentium*, (m) EPEC or (n) ETEC, nucleus stained with DAPI (blue).

To identify cytokines important for mucin production, we treated *in vitro* mucosal surfaces with polarized goblet cells, functional tight junctions, three-dimensional architecture and mucus secretion [31] with cytokines (Table 1) chosen based on increased expression accompanying increased mucus thickness (i.e. during clearance or differential expression in WT versus IFN-γ^−/-^ mice at 10 dpi). The cytokines were identified from a dataset of 85 genes involved in Th1/Th2 immune responses from a previous study using samples from the same mice [1]. The mucosal surfaces were treated with cytokines for 96 h to mimic the extended period of elevated cytokine stimulus occurring during *C. rodentium* infection *in vivo* [1]. In a first screen for the effects of the cytokines associated with increased mucus thickness on mucin production and goblet cell density, IL-6 and IL-12 did not induce changes; therefore we focused on IFN-γ, TNF-α, IL-4 and IL-13. In response to IFN-γ and TNF-α, the plumpness of the surface goblet cells and the overall morphology was reduced (p < 0.001), whereas IL-4 and IL-13 enhanced these features (p < 0.05, Figure 3(a-f)). Of these four cytokines, IFN-γ, TNF-α and IL-4 were present in WT animals during clearance, whereas IL-13 was only detected in IFN-γ^−/-^ mice [1].

To mimic infection *in vitro* is complex as bacteria divide fast and absence of mechanisms for eliminating bacteria result in high bacterial counts, which can be lethal to epithelial cells. The infecting doses were titrated to avoid detrimental effects on the epithelial cells, and yet, we could only accomplish this with a 24 h infection since longer experiments severely decreased the trans-epithelial resistance and killed the epithelial cells, regardless of the infecting dose. To achieve a viable co-culture, the infecting dose was 20-fold lower for *E. coli* than for *C. rodentium*, due to the slower growth of the latter. Bacteria were mainly present on the apical surface of the *in vitro* mucosa, although some were found between cells and basolaterally (Figure 3(l-n)). In the presence of *C. rodentium*, the cytokines had similar effects on the morphology and level of stored AB/PAS positive material in the tissue as in the absence of infection, although the responses to cytokines were slightly less pronounced compared to the non-infected membranes. EPEC and ETEC infection further blunted the response to the cytokines, although similar trends could be seen (Figure 3).

### *IL-4 protects the goblet cells and mucus production against the detrimental combined effect of IFN-γ, TNF-α and infection* in vitro

**Figure 4.**
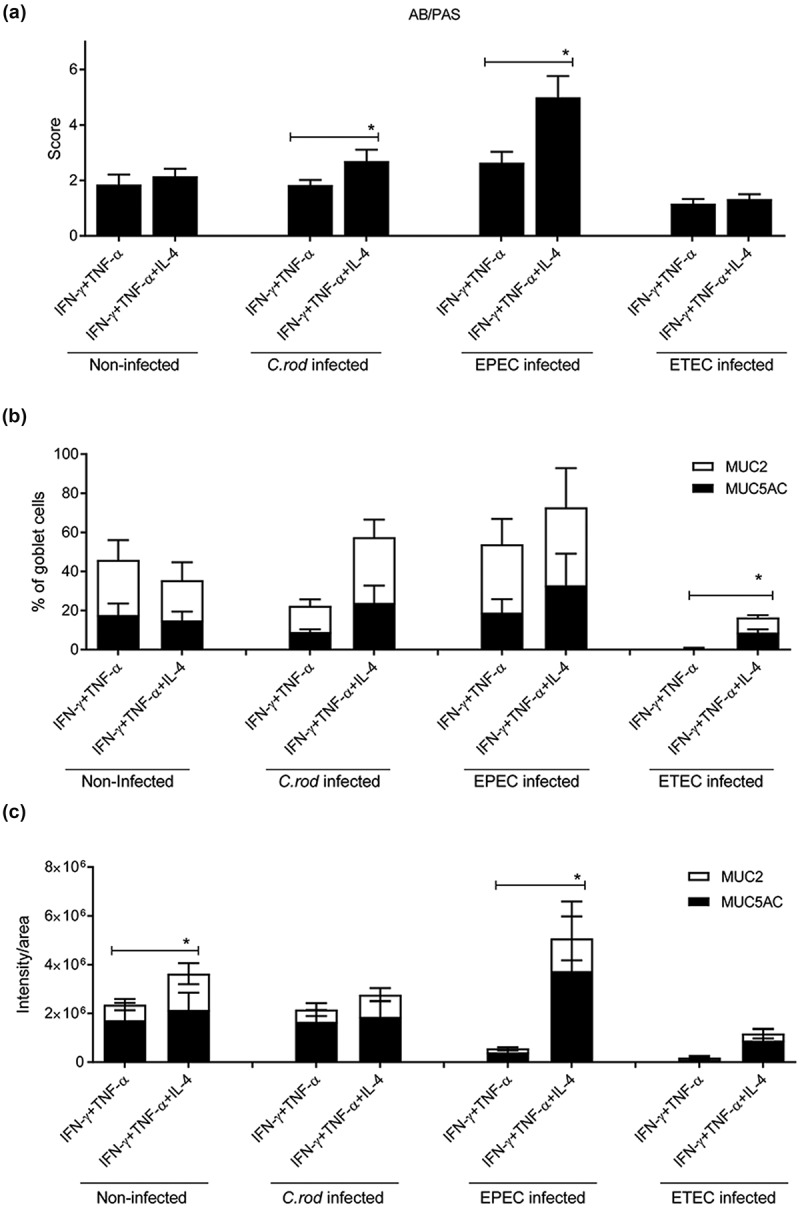
Combined IFN-γ and TNF-α *in vitro* treatment with and without addition of IL-4. The *in vitro* mucosal surfaces were cytokine treated for 4 days and infected for 24 h. (a) AB/PAS score: the score reflects the morphology (based on number of crypt like structures 0–4, integrity of the epithelial layer 0–4, and presence and mucus engorgement of surface goblet cells 0–4). (b, c) MUC2 and MUC5AC immunofluorescence; B) The percentage of the cells in the *in vitro* mucosal surfaces that were goblet cells was calculated in a blinded fashion. The white versus black parts of the bar corresponds to the proportion of the goblet cells that were positive for MUC5AC versus MUC2. Most goblet cells were positive for both mucins, although some only for one mucin. (c) Integrated fluorescence density obtained using the ImageJ program to quantify the mucins in the *in vitro* mucosal surface. Statistics: Wilcoxon signed rank test, *p < 0.05 (n = 3–5).

During *in vivo* clearance of *C. rodentium*, the epithelial cells are exposed to increasing levels of IFN-γ, TNF-α and IL-4 [1]. Treating the *in vitro* mucosal surfaces with IFN-γ and TNF-α simultaneously had severe effects on the morphology decreasing the mean score from 6 to 2 (compare Figure 4 with 3). The combined effects of IFN-γ and TNF-α were so severe that we lowered the concentration of each cytokine from 10 to 3 ng/ml, since the higher concentration caused cell death and tissue losses during processing. Although the mucus related parameter that was most affected differed between the pathogens, at least one mucus related parameter that was clearly improved by IL-4 treatment in each infection: in *C. rodentium* infected IFN-γ and TNF-α treated membranes the AB/PAS score increased by 50% (p < 0.05, Figure 4(a)), in EPEC infected ones the AB/PAS score doubled (p < 0.05, Figure 4(a)) and the intensity of the MUC2/MUC5AC stain increased 10-fold (p < 0.05, Figure 4(c)). In ETEC infected membranes the amount of goblet cells increased 24-fold (p < 0.05, Figure 4(b)). The differences between the infections are likely related to that the severity of the infection differed between pathogens or to inherent differences in host-pathogen interactions between the pathogens. The scenario most relevant to the in *vivo C. rodentium* infection is most likely that of EPEC, as this is an attaching and effacing pathogen interacting with cells from its natural host.

### In vitro mucin production and transport from Golgi to secretory vesicles at the apical surface is enhanced by IL-4 and infection

**Figure 5.**
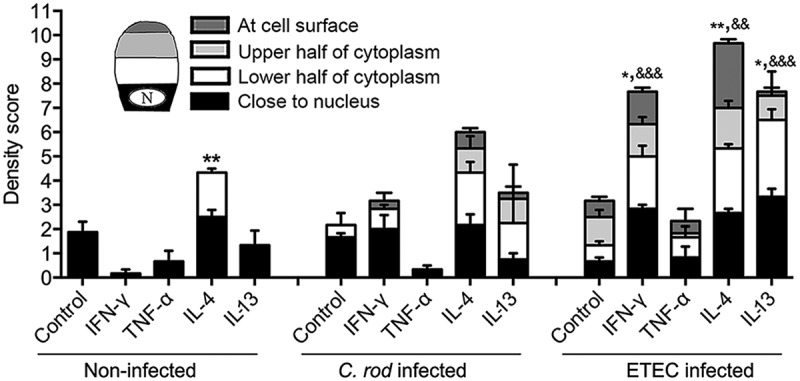
Mucin production and transport en route exocytosis in cytokine treated *in vitro* mucosal surfaces with and without infection. *In vitro* mucosal surfaces were harvested two h after addition of GalNAz to the basolateral media and the intensity and cellular location of incorporated label were detected by immunofluorescence. Scores for intensity and location of newly synthesized mucins were calculated as: close to nucleus (0–4), in the lower half of the cytoplasm (0–4), in the upper half of the cytoplasm (0–4) and at the cell surface (0–4) in a blinded fashion. Statistics: one way ANOVA, Dunnett’s post hoc test, *p < 0.05 and **p < 0.01 compared to control in each group, and one way ANOVA, Holm-Šídák’s post hoc test, &&p < 0.001 and &&&p < 0.0001 compared to the non-infected *in vitro* mucosal surfaces treated with the same cytokine (n = 3–4).

We compared incorporation and cellular localization of GalNAz labeled mucins at 2 h post addition of label (as a proxy for transport speed from Golgi to release at the apical surface), as the labeled mucins at that time mainly were inside the cells. In the absence of infection, addition of IFN-γ and TNF-α trended towards decreasing the amount of incorporated label, IL-13 had no apparent effect, while IL-4 increased the mucin production and transport speed en route exocytosis (p < 0.001, Figure 5). Although the effect of infection differed slightly between pathogens and which cytokine it was combined with, the overall effect of infection was increased mucin production and transport speed (p < 0.001). Infection with ETEC not only reversed the tendency to inhibition caused by IFN-γ, but enhanced the mucin transport (p < 0.05), and a similar trend was also found with *C. rodentium*. IL-4 and IL-13 increased the production further in the presence of infection (p < 0.05, Figure 5).

### IL-4^+^ T cells contribute to increased IL-4 expression and the mucus increase most likely occurs via the IL-4-receptor α/Stat6/Spdef pathway in vivo

The results above imply that IL-4 contributes to increased mucus thickness during clearance in WT mice, whereas both IL-4 and lL-13 may contribute to the increased thickness in IFN-γ^−/-^ mice at day 10 pi. To investigate the source of IL-4, we used flow cytometry for intracellular IL-4 staining on lymphocytes from the colon and MLNs. The frequency of IL-4^+^ cells in MLNs ranged from 0.5% to 1.4% for CD4^+^ T cells and 0.7% to 2.5% for CD8^+^ T cells, with no difference between the non-infected control animals and the *C. rodentium* infected animals. The frequencies of IL-4^+^ T cells in the colon were higher compared to MLNs (4.0% to 5.7% for CD4 and 4.3% to 6.5% for CD8, Supplementary Figure 3(a)), but again no difference was observed between the controls and infected animals. However, we observed a 4-fold increase in the T cell frequencies among live CD45^+^ cells in infected animals compared to the controls, and the larger absolute numbers of IL-4^+^ T cells in the infected animals could contribute to the increased IL-4 expression in the colon (Supplementary Figure 3(b)).

**Figure 6.**
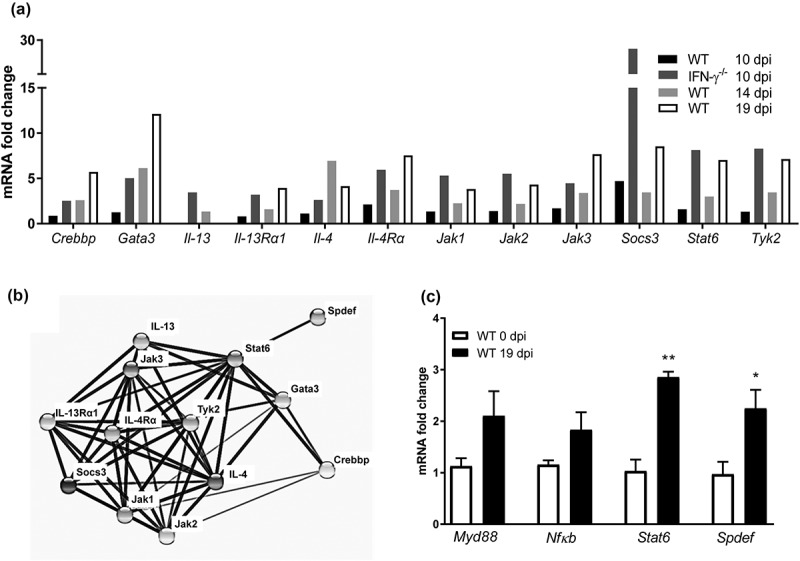
Expression of genes involved in mucin regulatory pathways during *C. rodentium* infection. (a) Changes in mRNA levels of infected WT (10, 14 and 19 dpi) and IFN-γ^−/-^ (10 dpi) mice, expressed as mean fold change compared to non-infected controls and based on a qPCR cytokine array containing 84 mouse inflammatory cytokines, chemokines and receptor genes. The data sets for time point 0 and 10 dpi represents four mice (pooled two and two) and the time points 14 and 19 dpi are representative of three mice. The differences between the data sets (biological and technical replicates) at each time point were less than 15%. Expression data was normalized against the *Gusb* housekeeping gene, which was calculated to be the most stable of the five existing housekeeping genes in the array. B) Interaction network of the proteins encoded in the mRNA array that were connected to Stat6, generated by the STRING v10database (https://string-db.org). The lines connecting each node were weighted for confidence of interaction based on experimental datasets and published information. Stronger associations are represented by thicker lines. (c) mRNA levels determined by qPCR in an additional three WT mice 0 and 19 dpi. Gene expression was normalized against *Hhprt* and *Eif2 *housekeeping genes. Fold changes were calculated using ∆∆CT with the mean CT from non-infected control mice. Statistics: unpaired t-test *p < 0.05 and **p < 0.01 (n = 3).

IL-13 induce airway mucus production via the IL-4-receptor α, Stat6 and the SAM pointed domain-containing ETS transcription factor, (Spdef) pathway [47,48]. An mRNA array demonstrated that *IL-4, IL-4-receptor α*, and *Stat6* transcription levels were increased both in the IFN-γ^−/-^ mice 10 dpi (but not in the WT mice at the same time), and in WT mice at 14 and 19 dpi (Figure 6(a)). Using the Search Tool for the Retrieval of Interacting Genes/Proteins database, we identified further Stat6 associated proteins among the ones that were upregulated in the array (Figure 6(a, b)), and finally qPCR on individual mice confirmed that *Spdef* and *Stat6* were upregulated during clearance of infection (p < 0.05, p < 0.01 Figure 6(c)). The toll-like receptor initiated pathway via *Myd88* and *NfκB* has also been described to induce airway mucus secretion, and these genes trended towards being upregulated during clearance (p = 0.13 and 0.12, Figure 6(c)). Thus, the *in vivo* increase in goblet cells, mucus production and transport rate potentially occur via the IL-4**/**IL-4-receptor α/Stat6/Spdef pathway, although other pathways may also contribute.

### In vivo manipulation of the IL-4/Stat6 pathway affects colitis, pathogen localization and mucus thickness and quality

**Figure 7.**
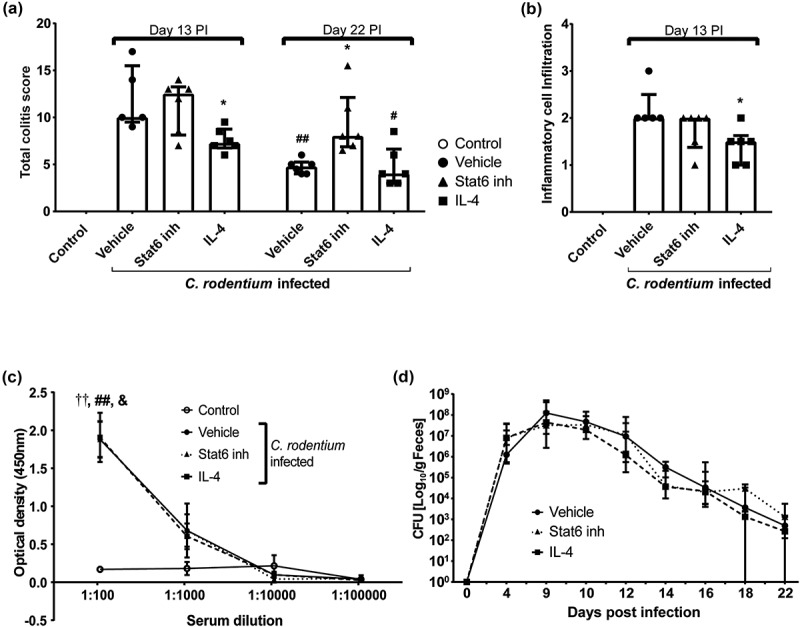
Effect of IL-4 and Stat6 inhibitor treatment on colitis, serum IgG and fecal CFU in *C. rodentium* infected WT mice. Mice received IL-4 (0.2 µg/mouse), Stat6 inhibitor (AS1517499, 10 mg/kg body weight [26]) or vehicle (sterile PBS containing 20% DMSO and 1% bovine serum albumin) by intraperitoneal injection for three consecutive days (for cohort 1: from day 11 to day 13, cohort 2: day 10 to day 12 pi). (a) Colitis scores for all treatment groups at 13 and 22 (clearance of infection ≤1000 CFUs/g feces) dpi. The score is the sum of crypt architecture, goblet cell depletion, leukocyte infiltration, presence of lamina propria neutrophils, crypt abscesses, and epithelial damage and ulceration. Results are shown from cohort 2 (n = 5–6/group and time point), and similar results were obtained from cohort 1 (n = 4–5/harvested day 14). (b) Inflammatory cell infiltration at 13 dpi (n = 5–6, cohort 2). (c) Mouse serum IgG response to *C. rodentium* determined by ELISA at 13 dpi (n = infected vehicle, Stat6 inhibitor and IL-4 treated 5–6, non-infected control 8). Statistics: Kruskall wallis test; *p < 0.05 vs vehicle, &p < 0.05 IL-4, ††p < 0.01 vehicle and ##p < 0.01 Stat6 inhibitor vs non-infected control. (d) Fecal *C. rodentium* CFU counts from infected groups: non-infected control mice were excluded as they did not have any *C. rodentium* colonies. Results shown are pooled from cohort 1 and 2 (n = 12–16/group until day 14 and 4–6/group until day 22). The analysis of fecal CFU counts was performed with a linear mixed effects model comparing the slopes of the three treatment groups (p = 0.44).

**Figure 8.**
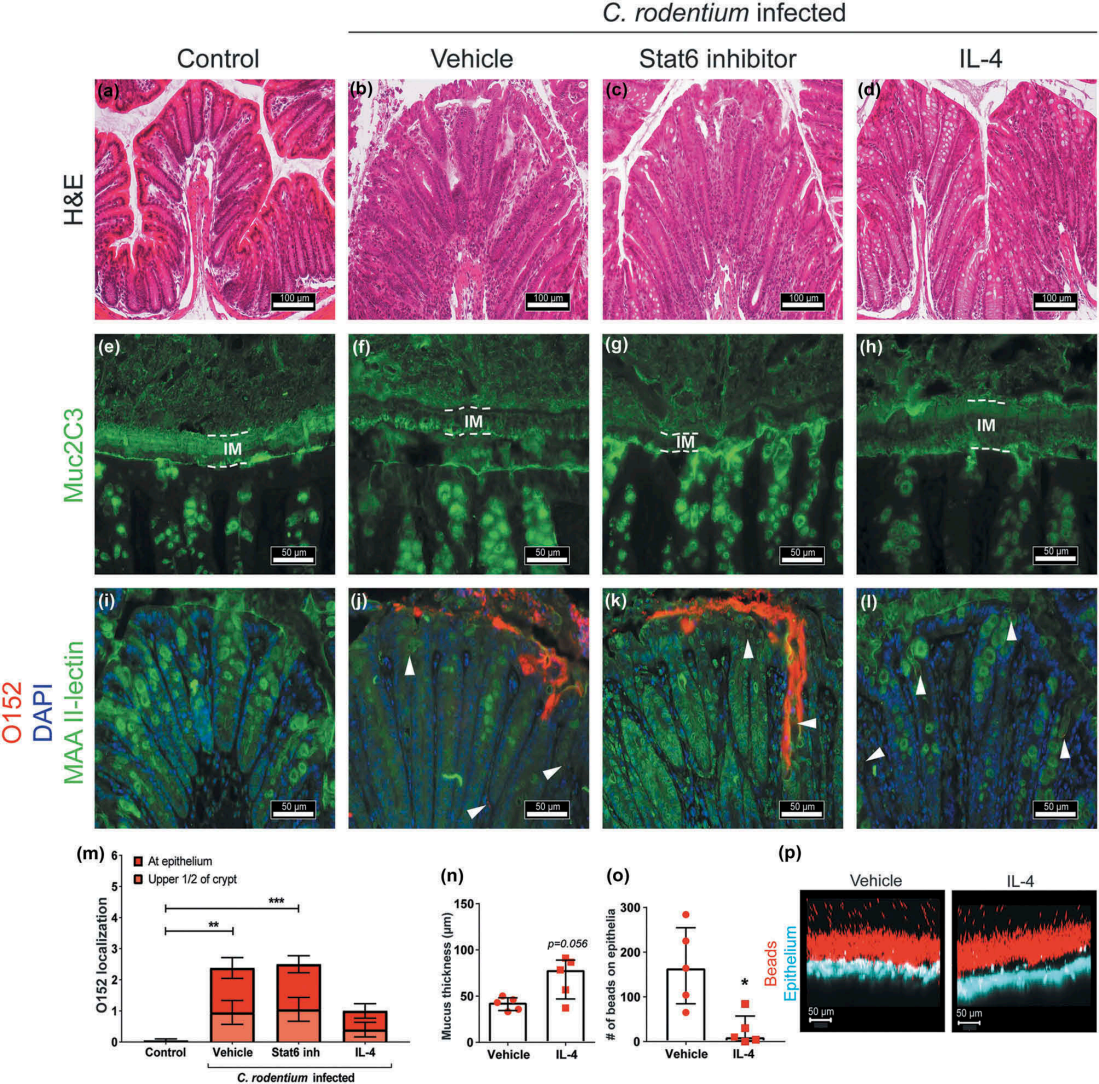
Effect of IL-4 and Stat6 inhibitor treatment on mucus thickness and quality and bacterial localization in WT mice. Representative images of H&E stained mouse distal colon samples from (a) non-infected non-treated, (b) infected vehicle, (c) infected Stat6 inhibitor and (d) infected IL-4 treated mice. Images were taken with a Nikon Eclipse 90i microscope, using a 20x objective. (e-h) Muc2 (green) immunofluorescence images showing (e) an organized, dense inner mucus layer (IM, highlighted in dotted lines) in non-infected non-treated control mice and (h) infected IL-4 treated mice, whereas F) the IM is poorly defined in infected vehicle and (g) Stat6 inhibitor treated mice at 13 dpi (n = 10, images representative for cohorts 2 and 1). (i-l) Localization of O152 positive bacteria (red) in distal colon (mucus stained with MAA II-lectin, green) in (i) non-infected control and (j) infected mice treated with vehicle, (k) Stat6 inhibitor or (l) IL-4 at 13 dpi (arrowheads point to bacteria). Images were captured using a 40x objective (n = 10, images representative for cohorts 2 and 1). (m) Quantification of bacterial localization in close association with the inner mucus layer/surface epithelial cells and crypts at 13 and 14 dpi (n = 10, results pooled from cohorts 1 and 2). N-P) *Ex vivo* imaging and analysis of mucus in the distal colon of infected vehicle and IL-4 treated mice using 1 µm beads; inner mucus layer thickness (n), penetrability of 1 µm sized beads (o) and representative images (1 µm sized beads: red, epithelium: blue) using an LSM700 confocal microscope at 20x objective (p) at 13 dpi (n = 5). Please note that mucus thickness in Figure 2 cannot be compared to the data presented here due to that the hydrophobic charcoal used for that measurement floats on top of the mucus, whereas the measurement of the inner mucus layer presented here use beads with ability to penetrate mucus with an altered structure. Statistics: (m) Kruskall wallis test; *p < 0.05, **p < 0.01, ***p < 0.001 vs non-infected control, and (n and o) Mann-Whitney U-test.

We initiated treatment with Stat6 inhibitor or IL-4 at 10 dpi to mimic a situation likely for a therapeutic regimen. All mice were colonized and soft stools were observed from day four pi onwards, with no clear difference between the treatment groups. *C. rodentium* infected mice treated with IL-4 for 3 consecutive days had lower colitis scores than vehicle or Stat6 inhibitor treated mice at 13 dpi (p < 0.05) and by 22 dpi the colitis scores of both vehicle (p < 0.005) and IL-4 (p < 0.05) treated mice had decreased, whereas the scores of Stat6 inhibitor treated mice remained high (Figures 7(a) and 8(a–d)). Furthermore, the level of inflammatory cell infiltration was lower in IL-4 treated mice compared with vehicle treated ones at 13 dpi (p < 0.05, Figure 7(b)). *C. rodentium* specific serum immunoglobulin G (IgG) increased to similar levels in all three infected treatment groups (p < 0.05, Figure 7(c)). The fecal CFUs decreased in all animals, regardless of treatment group: estimating the decrease of fecal CFU with a linear mixed effects model from day 10 to 18, the slopes were −0.58, −0.60 and −0.50 log units/day for vehicle, IL-4 and Stat6 inhibitor treated groups, respectively (Figure 7(d)). Furthermore, the amount of *E. coli* LPS O152 positive bacteria (also present in *C. rodentium*) in close association with the epithelium and in the inner mucus layer was abundant in infected vehicle and Stat6 inhibitor treated mice (p < 0.01), whereas they were almost absent in these locations in IL-4 treated mice and not statistically different from the non-infected mice at 13 dpi (Figure 8(e-m)). Although very few O152 positive bacteria were present in association with the epithelial cells or the inner mucus layer in the non-infected mice (Figure 8(i–m)), we detected O152 positive bacteria in the fecal pellet (Supplementary Figure 4(e, i and m). In non-infected mice, O152 negative bacteria formed large aggregates and dominated the fecal flora, but at 13 dpi these large aggregates were absent in all infected mice, regardless of treatment, although O152 negative bacteria remained present. O152 positive bacteria were present at a high density in feces from vehicle and Stat6 inhibitor treated infected mice whereas a low density was observed in feces from IL-4 treated mice (Supplementary Figure 4), analogous with the tendency to fewer *C. rodentium* detected by fecal CFU counts in these mice.

In contrast to vehicle or Stat6 inhibitor treated mice, the mucus layer in IL-4 treated mice appeared thick and organized in a similar fashion as in the non-infected mice (Figure 8(e-h)). *Ex vivo* live imaging of mucus on colonic mucosa supported that infected IL-4 treated mice had a mucus layer thicker than vehicle treated infected mice at 13 dpi (p = 0.056, Figure 8(n, p)). Although the mucus allowed bacteria-sized fluorescent beads to penetrate, the function of the mucus in infected IL-4 treated mice was better than in vehicle treated mice since more beads reached the epithelium in vehicle treated infected mice (p < 0.05, Figure 8(o, p)).

## Discussion

In the present study, we revealed that changes in the cytokine environment induce increased production and transport of mucins from the Golgi to the secretory vesicles at the apical surface during clearance of infection. During *C. rodentium* infection, IFN-γ^−/-^ mice had similar pathogen burden, but different cytokine environment, 4-fold thicker mucus layer and more stored mucin in the tissue than WT mice, demonstrating that the cytokine environment impacts mucus thickness during intestinal infection *in vivo*. Neither the enhanced mucus thickness found during clearance in the WT mice [41], nor the increased mucus thickness observed in the IFN-γ^−/-^ mice during infection could be explained by changes in mucin mRNA levels. Instead, experiments on *in vitro* mucosal surfaces showed that the local cytokine environment, namely IL-4, and infection have major effects on mucin production and transport speed. Increased expression of *Il-4, Il-4-receptor α, Stat6* and *Spdef*, indicate that this pathway may be responsible for the increase in mucus production and transport speed, and manipulating this pathway affected mucus thickness and quality as well as the amount of bacteria in close contact with the host epithelial cells and colitis levels.

IL-4 is produced by T cells, mast cells and basophils and has many biological roles in innate and adaptive immunity. Of particular relevance to the current study, IL-4 contributes to increased antibody responses after infection [49]. In the airways, IL-13 has been shown to induce mucus production via the IL-4-receptor α, Stat6 and Spdef pathway [47,48]. This pathway has been implicated in healing, but with contrasting results: IL-4 has been demonstrated to impair wound healing in the skin [50], whereas Stat6^−/-^ mice display delayed wound healing in mice treated with 2,4,6-trinitrobenzenesulfonic acid [51]

We have previously shown that mucin production and vesicle transport rate decrease during acute and chronic infection with *Helicobacter pylori* in the murine stomach [52]. Similar to *C. rodentium, H. pylori* is associated with a Th1/17 cytokine response, but in contrast, *H. pylori* causes a chronic infection associated with reduced expression of IL-4 [53]. When the mucus thickness increases, concomitantly with the increase in mucin production and transport during clearance of *C. rodentium*, the environment is no longer of a typical Th1/17 cytokine profile, but also contains Th1/17-opposing cytokines, including IL-4.

*In vitro*, IL-4 and IL-13 generally had positive effects on mucin production and transport, whereas IFN-γ and TNF-α had negative effects, although the negative effects were partially counteracted by the presence of pathogens, demonstrating a concerted regulation of these events. In a previous study using an allergic model, Th2 cells intravenously transferred into mice resulted in high levels of IL-4 and increased levels of AB/PAS stained mucins in the tissue [54]. In contrast, transfer of twice the number of Th1 as Th2 cells decreased the amount of AB/PAS stained mucins in an IFN-γ receptor dependent manner [7]. Although these results only depict the level of mucin in tissue, they are in line with the thinner mucus layer and lower amount of stored mucin in the colon of the infected WT mice compared to IFN-γ^−/-^ mice found in this study. However, in *C. rodentium* infected WT mice, an increase in the mucus thickness occurs from 14 dpi throughout 19 dpi [41]. This stands in contrast to IFN-γ as a negative regulator of mucus thickness since we also observed a successive increase in *Ifn-γ* mRNA throughout clearance of the pathogen. This increase in mucus layer thickness starts after 10 dpi, which is the time point when the bacteria reach the highest density in the lumen, mucus thickness reduced to its lowest level, and an organized striated inner mucus layer was rarely found [41]. The absence of a protective barrier as well as high bacterial density likely lead to that an abundance of bacterial products reaches the epithelial cells. Indeed, *in vitro* infection in combination with IFN-γ enhanced mucin production and transport through the cell en route exocytosis, in contrast to the decrease caused by IFN-γ alone. Therefore, the bacterial stimulation together with the concomitant increase of IL-4 and IFN-γ are likely the reasons for the increased mucus thickness during *C. rodentium* clearence. *Tnf-α* was up-regulated during the clearance phase of infection, coinciding with an enhanced mucus layer (14 and 19 dpi) in WT mice. Treating the *in vitro* mucosal surfaces with this cytokine had similar effects on mucus production as IFN-γ, decreasing the number of goblet cells, mucus thickness and transport. Furthermore, the combination of IFN-γ and TNF-α damaged the integrity of the epithelial surface and reduced the number of goblet cells. This damaging effect is in line with the result of a study on the C1.16E cell line cultured as a monolayer that showed treatment with these cytokines flattens the cells and devoid them of mucus granules [9]. Although there are sloughed off goblet cells present throughout infection and clearance *in vivo* [13], the presence of IL-4 appears to be a factor limiting the negative effects of these cytokines, and indeed IL-4 has previously been shown to protect the mucosal mitochondrial activity during *C. rodentium* infection [1].

Of the cytokines upregulated during times of an enhanced mucus layer, IL-4 was the most efficient enhancer of goblet cell numbers, surface goblet cell engorgement, as well as production and transport rate of mucin vesicles through the cell. This is in contrast to worm (*Trichinella spiralis*) infection, during which IL-13, but not IL-4, is affecting murine goblet cell proliferation [6], pointing to that the combined effect of the pathogens and the immune system determines the mucin/mucus changes during infection. IL-13 may however contribute to the vastly increased mucus thickness in the IFN-γ^−/-^ mice at 10 dpi, as both IL-4 and IL-13 increased in these mice. In addition to enhancing mucin production and transport, IL-4 also had a protective effect on the integrity of the *in vitro* mucosal surface, goblet cell number, and the amount of mucin produced during infection. Increased levels of *Il-6* mRNA occurred both in *C. rodentium* infected IFN-γ^−/-^ mice at day 10 pi, and in the WT mice at day 14 and 19 pi, coinciding with the increase in mucus thickness in both systems. Although IL-6 in other contexts has been shown to phosphorylate Stat6 [55], addition of IL-6 alone had no effect on any of the measured mucus related parameters in the *in vitro* mucosal surfaces. Previous studies have shown that IL-6 contributes to polarization of naive CD4^+^ T cells to effector Th2 cells which then initiate production of IL-4 [56]. Thus, IL-6 may enhance the mucin production via induction of IL-4 *in vivo*.

Together, the data suggests that the *in vivo* increase in mucus production and transport speed may occur via the IL-4/IL-4-receptor α/Stat6/Spdef pathway, and manipulating this pathway affects mucus penetrability, thickness, and pathogen localization *in vivo*. Although members of this pathway affect functions in B cells, T cells and macrophages, and the effect on bacterial location thus cannot be certified to solely depend on the increase in mucus production and transport, these results support the *in vitro* data that this pathway plays an important role during clearance of infection. Mucolytic agents have bactericidal and antioxidative effects [57,58], making it difficult to prove that the increase in mucus production is the only cause for the decreased pathogen burden in IL-4 treated mice. However, *C. rodentium* infection in mice lacking Muc2 results in high mortality [11], mice with defective mucus exocytosis display delayed *C. rodentium* clearance [12] and *C. rodentium* bind to murine Muc2 [13]. These findings, together with our observation that IL-4 treatment increase mucus thickness and quality and decrease colitis and the number of pathogens in contact with the epithelium, demonstrate that mucus secretion is important for *C. rodentium* clearance. B-cell deficient mice infected with *C. rodentium* fail to clear the infection and IgG is important for infection clearance [14,15]. However, the amount of *C. rodentium* specific serum IgG was similar in infected mice regardless of IL-4, Stat6 inhibitor or vehicle treatment, suggesting that the effects seen in this study were not due to changes in IgG levels. Furthermore, IL-4 has previously been shown to inhibit macrophage killing of *C. rodentium* [59], suggesting that the IL-4 treated mice would be at a disadvantage, if the epithelial cell response was not of importance. The microbiota has been shown to be important for clearance of *C. rodentium* infection, since germ free mice do not clear the infection, but addition of commensals aid in removal of the infection/pathogen when added after the normal clearance time [60]. Since germ free mice have a thin disorganized mucus layer [61], these results are compatible with a clearance model where IL-4 dependent mucus and defensive factors secreted into the mucus aid in removing the pathogen from the epithelial surface, and once the pathogen is in the lumen, the recovering commensal flora aids by outcompeting the pathogen. The beneficial role of IL-4 is in line with a study demonstrating a correlation between increased IL-4 levels and short duration of ETEC infection [62]. Furthermore, IL-4 administration had beneficial effects in both animal houses used in this study. Although the facilities used were relatively similar, some factors, such as the microflora, are likely to differ. This suggests that the effect of IL-4 is of general nature.

The IL-4 treatment started at height of infection and three days of treatment led to that the pathogen was removed from the colonic epithelial surface earlier than in vehicle or Stat6 inhibitor treated mice and this was accompanied by a 30% decrease in colitis. Prophylactic rather than therapeutic treatments generally lead to more pronounced effects, however, the latter is more relevant from a clinical perspective. The removal of the pathogen from the epithelial surface and recovery of a functional mucus layer is more important for healing than the presence of pathogen in the fecal pellet as pathogens can be present in the lumen for long periods without causing symptoms and can remain for months after recovery from an infectious disease. Indeed, both in murine colitis models and ulcerative colitis, bacterial penetration of the normally impenetrable mucus layer and access to the epithelium are important determinants of colitis [3]. In addition to colitis, ETEC and EPEC infection also cause diarrhea. The mouse strain used in the current study did not develop watery diarrhea but instead, semi-solid fecal pellets were observed with the infectious dose used here. A more susceptible mouse model, such as FVB/N might be more appropriate to study the effect of treatments on diarrhea [63].

In conclusion, our results demonstrated that the cytokine profile and pathogen species act in concert to impact several parameters that affect mucus thickness. IL-4 was the cytokine that demonstrated most beneficial effects, both with regards to mucin production parameters, and also in that it protected epithelial surface integrity *in vitro* against the detrimental effects of IFN-γ, TNF-α and the presence of pathogens. Thus, during clearance of infection, the increase in IL-4 maintains goblet cell function and protects it against the increasing levels of TNF-α and IFN-γ. Furthermore, the IL-4/Stat6 pathway is an important determinant of intestinal mucus production that can be manipulated and affect bacterial localization. Manipulating this pathway may thus be a therapeutic option to enhance healing of the mucosa and shorten the duration of infections, or to induce healing during chronic infections.

## Abbreviations

*C. rodentium*: *Citrobacter rodentium*
*E. coli*: *Escherichia coli*
ETEC: Enterotoxigenic *Escherichia coli*
EPEC: Enteropathogenic *Escherichia coli*
EHEC: Enterohaemorrhagic *Escherichia coli*
A/E: Attaching and effacing
*H. pylori*: *Helicobacter pylori*
LPS: Lipopolysaccharide
CFU: Colony forming unit
MLN: Mesenteric lymph nodes
IgG: Immunoglobulin G
Th: T helper cell
dpi: Days post infection
WT: Wild type
IL: Interleukin
IFN-γ: Interferon gamma
IFN-γ^-/-^: Interferon gamma deficient
TNF-α: Tumor necrosis factor alpha
Stat6: Signal transducer and activator of transcription 6
Spdef: SAM pointed domain containing ets transcription factor
RCM-1: Robert Costa Memorial drug-1
*Myd88*: Myeloid differentiation primary response 88
*NFκb1*: Nuclear factor kappa B subunit 1
*Gusb*: Glucuronidase beta
*Hprt*: Hypoxanthine Guanine Phosphoribosyltransferase
*Hsp90ab1*: Heat Shock Protein 90 Alpha Family Class B Member 1
*Gapdh*: Glyceraldehyde-3-Phosphate Dehydrogenase
*Actb*: Actin beta
GalNAz: N-acetylgalactosamine
TAMRA: Tetramethylrhodamine
DMSO: Dimethyl sulphoxide
BSA: Bovine serum albumin
FBS: Fetal bovine serum
PD: Transepithelial potential difference
AB: Alcian blue
PAS: Periodic acid Shiff
H/E: Haematoxylin/eosin
MAA ІІ: Maackia amurensis ІІ
ELISA: Enzyme-Linked Immunosorbent Assay
OD: Optical density
EDTA: Ethylenediaminetetraacetic acid
LC–MS: Liquid chromatography–mass spectrometry

## Ackknowledgments

This work was supported by the Swedish research council Formas (221-2011-1036 and 221-2013-590), the Swedish Cancer Society, the Ragnar Söderberg, RR Julin, Jeansson and WM Lundgren Foundations. The funders had no role in study design, data collection and analysis, decision to publish, or preparation of the manuscript.
