# Supplementary material for: Interleukin 4 induces rapid mucin transport, increases mucus thickness and quality and decreases colitis and Citrobacter rodentium in contact with epithelial cells
Source: Virulence. 2019 Jan 21;10(1):97–117. doi: 10.1080/21505594.2019.1573050 (PMC6363059; doi:10.1080/21505594.2019.1573050)
Supplement: Supplemental Material [file kvir-10-01-1573050-s001.zip › Supplementary Legends.docx]

**Supplementary Figure 1. Representative H/E images of WT and IFN-γ^-/-^ mice.**

H/E staining of WT (A) and IFN-γ**^-/-^** (B) mice infected with *C. rodentium* at day 10 pi. Images were taken with a Nikon Eclipse 90i microscope, using a 20x objective.

**Supplementary Figure 2.** **Mucin mRNA levels in the distal colon of WT and IFN-γ^-/-^ mice at day 10 pi.**

*Muc1* (A), *Muc2* (B), *Muc4* (C), *Muc13* (D) and *Clca-3* (E). Expression data were normalized against the *Hprt-1* housekeeping gene. Fold changes were calculated using ΔΔCT with the mean CT values from three non-infected mice as controls. *Muc5AC* and *Muc6* mRNA were not expressed in either genotype at any time point. Statistics: one way ANOVA, Holm-Šídák’s post hoc test, #p<0.05, ##p<0.001 vs day 0 of the WT mice, *p<0.05, **p<0.001 vs day 0 of the IFN-γ**^-/-^** mice (n = IFN- **γ ^-/-^** day 0: 4, day 4: 3, and day 10: 5, WT: 5).

**Supplementary Figure 3. Identification of T-cells as a source of IL-4 during *C. rodentium* infection.** (A) IL-4 production by colonic T cells: A representative flow cytometry analysis of cells from *C. rodentium* infected murine colonic lamina propria lymphocytes stimulated with PMA/ionomycin is shown (n = 2 pooled two and two)**.** Cells were gated on singlet, live CD45^+^ cells and further subdivided into TCRβ^+^CD4^+^ and TCRβ^+^CD8^+^ cells. B) T cell frequencies (among live CD45^+^ cells) in non-infected vs *C. rodentium* infected murine colonic lamina propria lymphocytes.

**Supplementary Figure 4. *E. coli* lipopolysaccharide O152 antigen positive and negative eubacteria in fecal pellets.**

Colonic tissue sections were co-stained with a general eubacterial probe (Eub338, red) and an antibody against *E. coli* lipopolysaccharide antigen O152 (green), which also stains *C. rodentium.* Images of fecal material were captured in the red channel (A-D), in the green channel (E-H), merged (I-L), and the white rectangle indicated in I-L at a higher magnification (M-P) from non-infected non-treated and vehicle, Stat6 inhibitor and IL-4 treated mice at 13 dpi (n = 5-6). Arrowheads highlight large eubacterial colonies found only in non-infected mice.
