# Supplementary figures and images for: Interleukin 4 induces rapid mucin transport, increases mucus thickness and quality and decreases colitis and Citrobacter rodentium in contact with epithelial cells
Source: Virulence. 2019 Jan 21;10(1):97–117. doi: 10.1080/21505594.2019.1573050 (PMC6363059; doi:10.1080/21505594.2019.1573050)

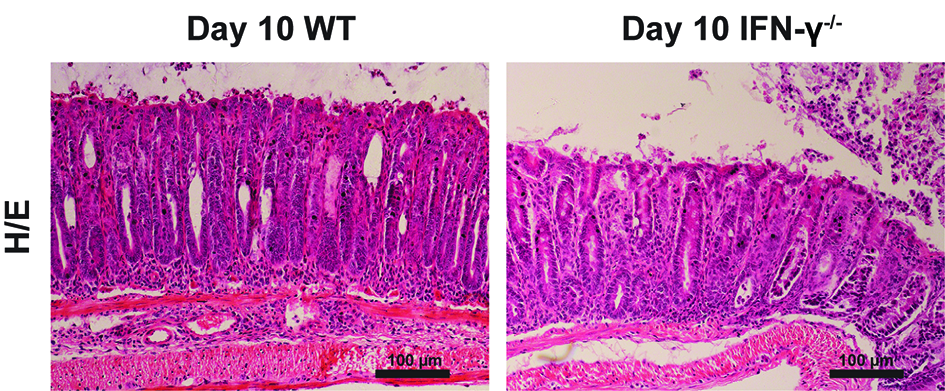

Supplement: Supplemental Material [file kvir-10-01-1573050-s001.zip › Supplementary Figure 1.tif]

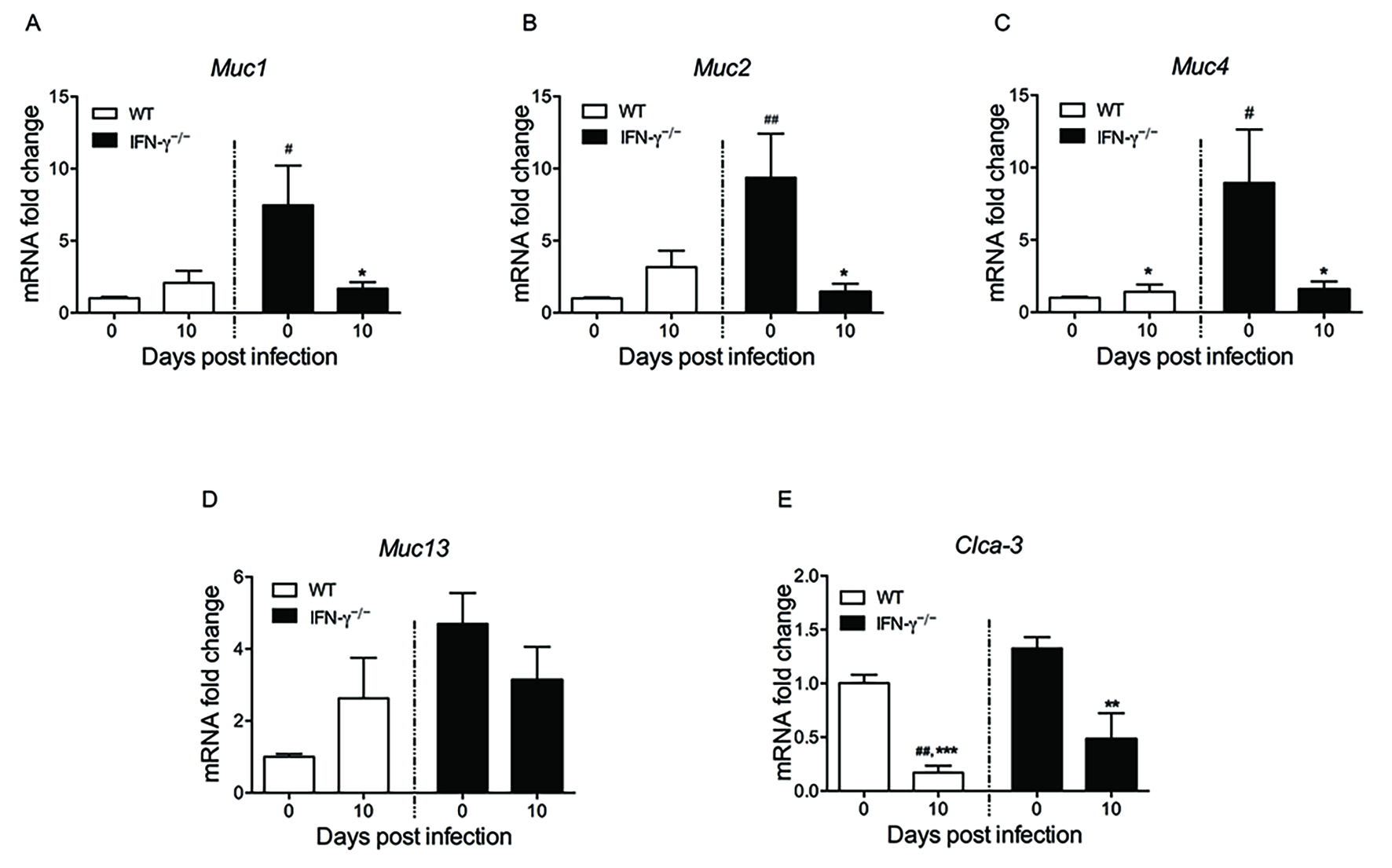

Supplement: Supplemental Material [file kvir-10-01-1573050-s001.zip › Supplementary Figure 2.tif]

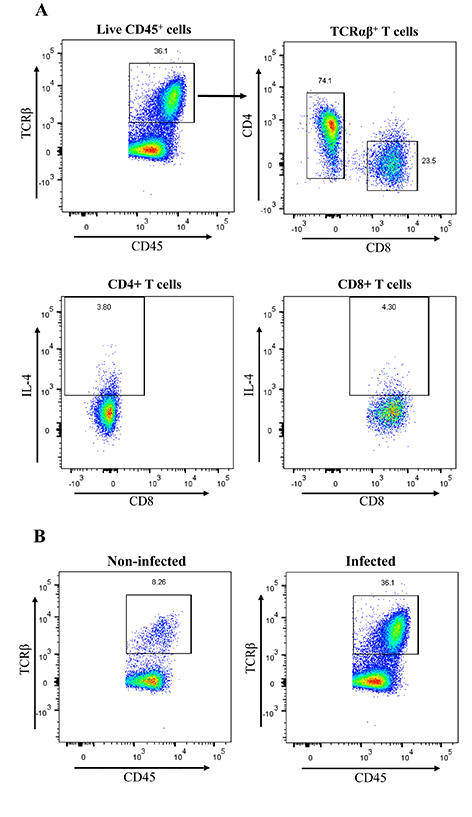

Supplement: Supplemental Material [file kvir-10-01-1573050-s001.zip › Supplementary Figure 3.tif]

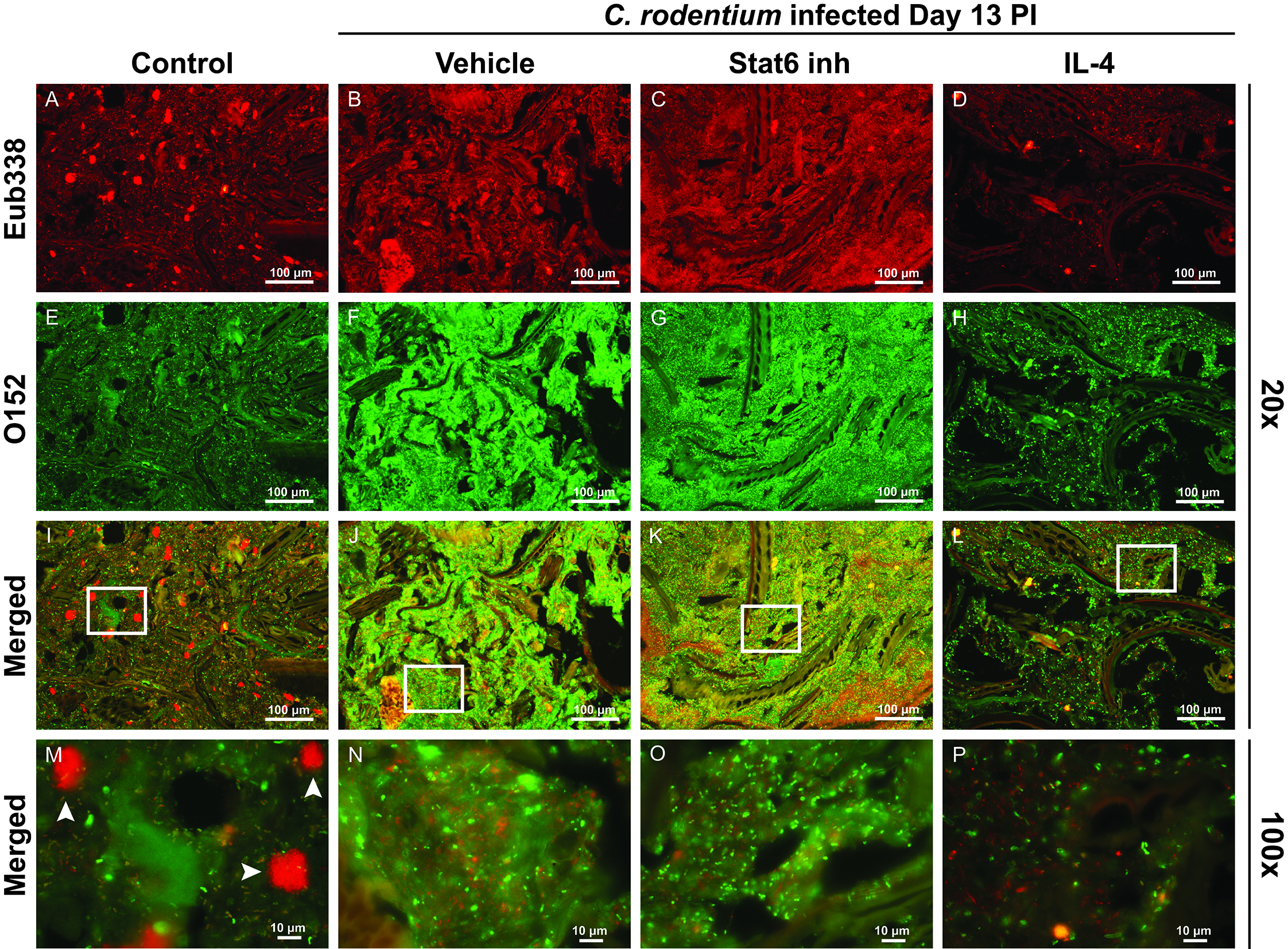

Supplement: Supplemental Material [file kvir-10-01-1573050-s001.zip › Supplementary Figure 4.tif]
